# Synthesis of benzo[*f*]quinazoline-1,3(2*H*,4*H*)-diones

**DOI:** 10.3762/bjoc.20.228

**Published:** 2024-10-28

**Authors:** Ruben Manuel Figueira de Abreu, Peter Ehlers, Peter Langer

**Affiliations:** 1 Universität Rostock, Institut für Chemie, Albert-Einstein-Str. 3a, 18059 Rostock, Germany

**Keywords:** cross-coupling, cyclization, heterocycles, palladium

## Abstract

We report the synthesis of polycyclic uracil derivatives. The method is based on palladium-catalysed Sonogashira–Hagihara and Suzuki–Miyaura cross-coupling reactions followed by Brønsted acid-mediated cycloisomerisation. The developed methodology tolerates various functional groups and leads to moderate up to quantitative yields of the final products. The impact of different functional groups on the optical properties was studied by UV–vis and fluorescence spectroscopy.

## Introduction

**Figure 1:**
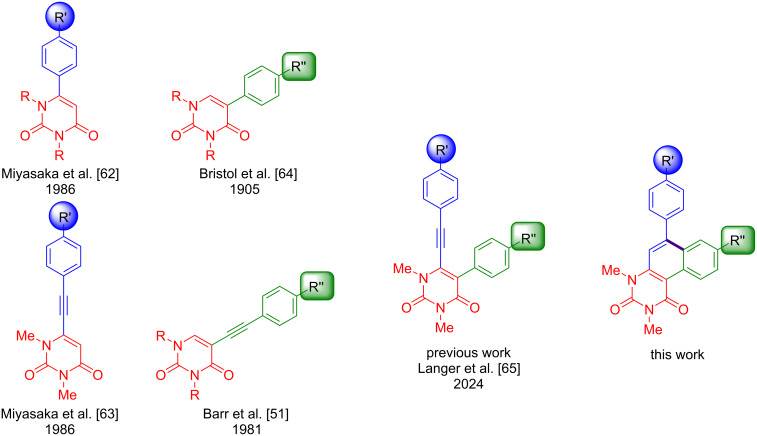
Synthesis of uracil-based alkynes and aryl structures [51,62–65].

Nucleobases contain the coded information and give DNA and RNA their typical structure. As a nucleobase, uracil is involved in numerous vital processes and is therefore a promising target and candidate for the development of new drugs against a wide range of diseases [1–4]. As it is not contained in the DNA, it could be used to distinguish between DNA and RNA-based pharmaceutical targets. In previous years, uracil has been successfully used in the development of several drugs that are still important and often used. Examples include 5-fluorouracil and brivudine. 5-Fluoruracil is an important anticancer drug, while brivudine is considered to be one of the most effective antiviral drugs against the HSV-1 virus [4–20]. Consequently, in recent years, new methods have been developed to introduce various functional groups at position 5 or 6 of uracil [19–50]. However, only a few methods are known which allow for an individual introduction of substituents at both positions [37–38,51–61]. In our previous work, we developed a new method which enables both positions to be independently functionalised by Sonogashira- and Suzuki–Miyaura cross-coupling reactions (Figure 1).

**Figure 2:**
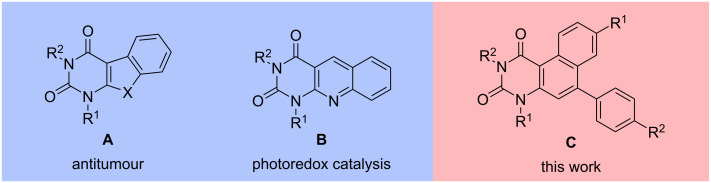
Structures of uracil derivatives **A**, **B**, and **C**.

Polycondensed heterocycles containing a uracil moiety have also been studied in recent years. For example, compound **A** exhibits antitumor and antimicrobial properties (Figure 2) [66–67]. Compounds **A** and **B** are used as starting materials for the synthesis of polyaromatic derivatives of other compounds with medicinal or photophysical properties [52–55]. Compounds **A** are related to the class of natural products known as coumestans, while **B** resembles flavins. While the medical potential of coumestans is still the subject of current research, interesting photocatalytic properties have already been identified for flavins, making them interesting for photoredox catalysis [68–69]. Inspired by the current interest in the synthesis of novel uracil-derived cyclic compounds and our previous studies, we herein wish to report a new method for the synthesis of a series of novel uracil-based benzo[*f*]quinazoline-1,3(2*H*,4*H*)-diones **C** [65]. Furthermore, optical properties were analysed by UV–vis and fluorescence spectroscopy.

## Results and Discussion

### Synthesis

**Scheme 1:**
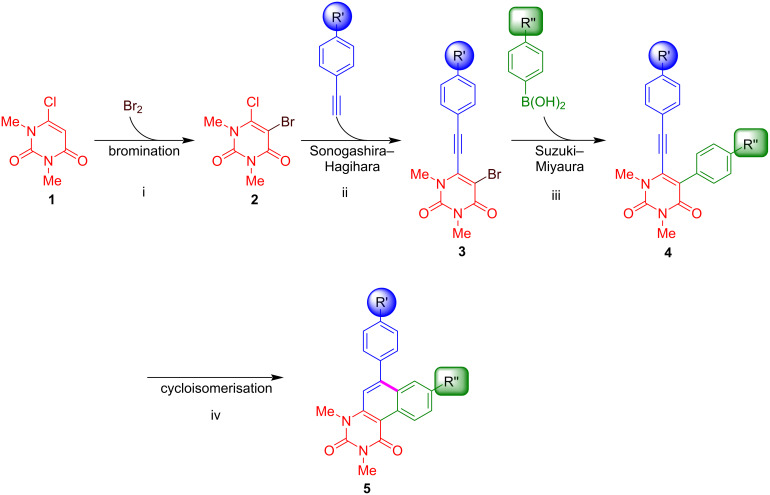
Strategy for the synthesis of the cyclised product **5**. Conditions: i) Br_2_ (2 equiv), Ac_2_O (1.5 equiv), AcOH, 25 °C, 1 h [65]; ii) Pd(PPh_3_)Cl_2_ (5 mol %), CuI (5 mol %), NEt_3_ (10 equiv), DMSO, 25 °C, 6 h; iii) Pd(PPh_3_)_4_ (10 mol %), NaOH (3 equiv), 1,4-dioxane/water 5:1, 100 °C, 1 h; iv) *p*-TsOH (20 equiv), toluene, 100 °C, 4 h.

**Scheme 2:**
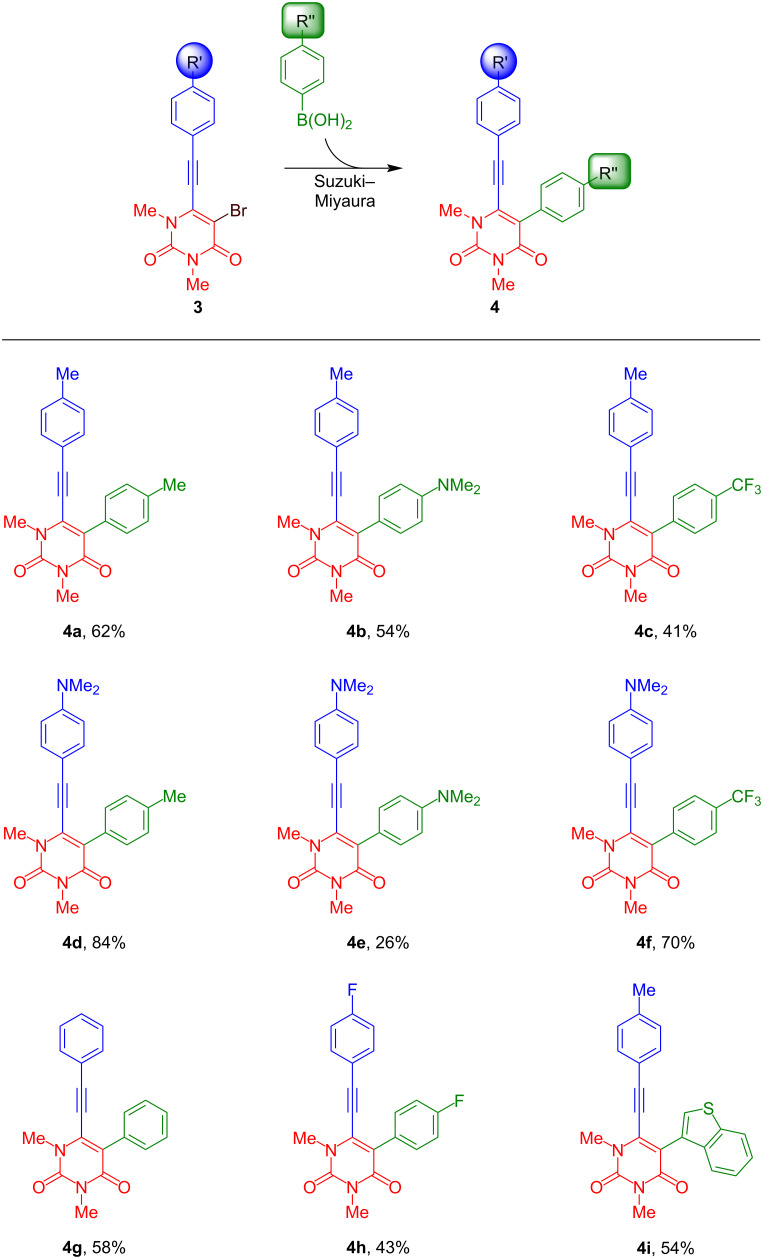
Synthesis and isolated yields of 1,3-dimethyl-5-aryl-6-[2-(aryl)ethynyl]uracils **4a–i**. Reaction conditions: **3** (1 equiv), boronic acid (1.2 equiv), Pd(PPh_3_)_4_ (5 mol %), NaOH (3 equiv), 1,4-dioxane/water 5:1, 100 °C, 1 h.

Our strategy for the synthesis of benzo[*f*]quinazoline-1,3(2*H*,4*H*)-diones is based on a four-step sequence and relies on a combination of palladium-catalysed Sonogashira–Hagihara and Suzuki–Miyaura cross-coupling reactions (Scheme 1). The final cyclisation step is accomplished by an acid-mediated cycloisomerisation. The synthesis of starting materials **4** was carried out by our previously reported protocol [65]. While compounds **4a–f** are known compounds, the synthesis of derivatives **4g**–**i** has not been previously reported (Scheme 2). Yields were generally lower in case of the presence of electron-withdrawing substituents.

Subsequently, the cyclisation of **4a–i** to **5a–i** by acid-mediated cycloisomerisation was studied. In our first attempt, we used platinum(II) chloride (PtCl_2_) as the Lewis acid and **4a** as the model starting material. The reaction afforded a mixture of the desired product **5a** and the starting material. Separation of both compounds proved to be difficult and, hence, optimisation was carried out as depicted in Table 1. The employment of higher temperatures resulted again in a mixture of **4a** and **5a** (Table 1, entry 2). Next, *p*-toluenesulfonic acid (*p*-TsOH·H_2_O) was chosen as the Brønsted acid. To our delight, product **5a** was obtained in a yield of 59% after 2 hours (Table 1, entry 3). Further investigation showed that the yield could be improved to 99% by increasing the reaction time to 4 hours (Table 1, entry 4), while reduction of the amount of acid resulted in a drop of the yield to 85%.

**Table 1:** Optimization of the synthesis of **5a**.

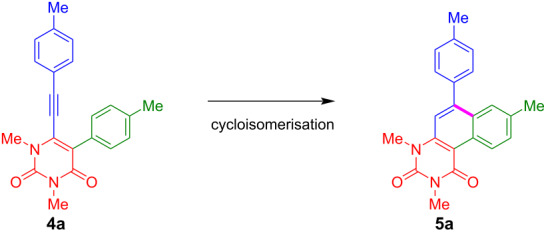					

Entry	Acid (equiv)	Solvent	Temperature (°C)	Time (h)	Yield (%)

1	PtCl_2_ (0.1)	toluene	80	15	mixture
2	PtCl_2_ (0.1)	toluene	100	15	mixture
3	*p*-TsOH·H_2_O (20)	toluene	100	2	59
4	*p*-TsOH·H_2_O (20)	toluene	100	4	99
5	*p*-TsOH·H_2_O (15)	toluene	100	4	85

**Scheme 3:**
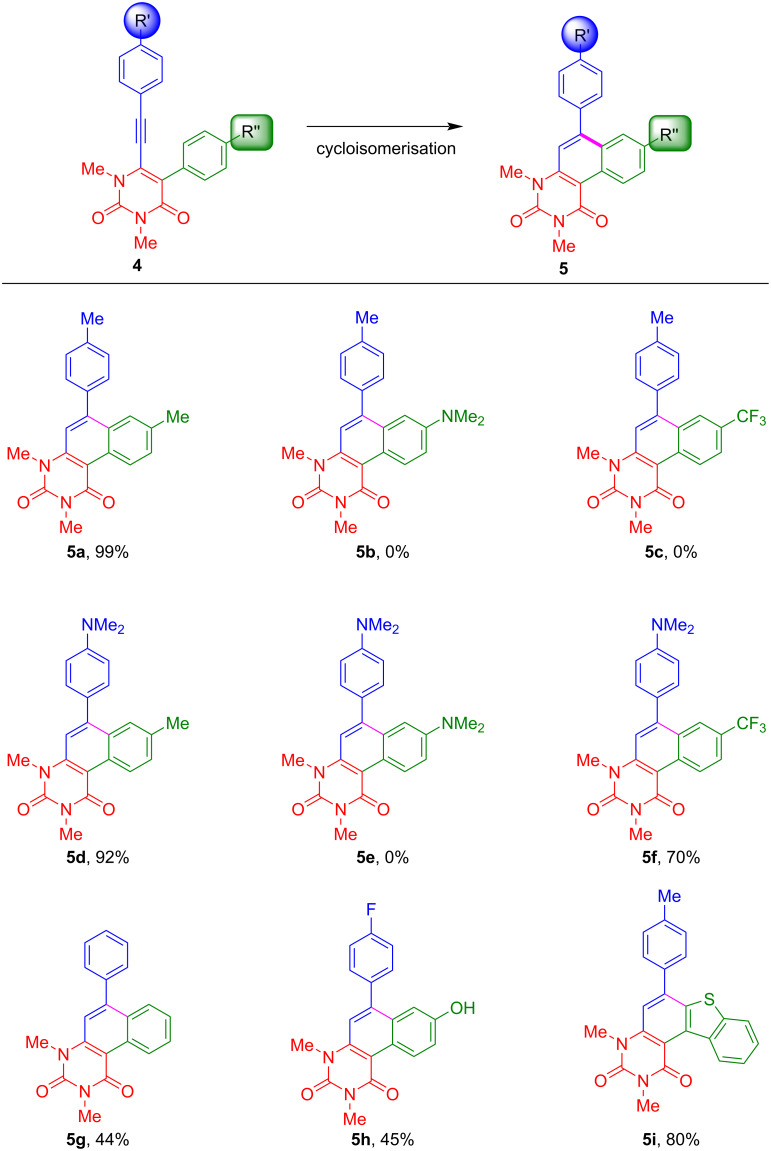
Scope and isolated yields of the synthesis of **5**. Reaction conditions: **4** (1 equiv), *p*-TsOH·H_2_O (20 equiv), toluene, 100 °C, 4 h.

**Scheme 4:**
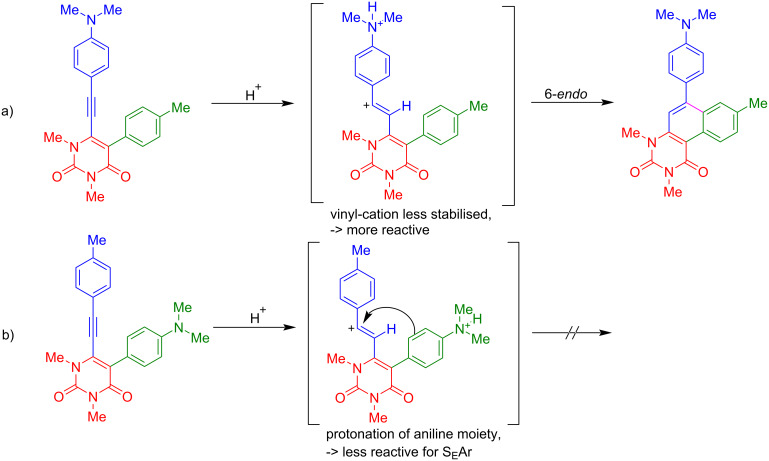
Proposed reaction mechanism of the cyclisation with *N,N*-dimethylanilino functional groups.

With the optimised conditions in hand, the scope of the cycloisomerisation was studied and the products **5a**, **5d**, and **5f–i** were isolated in moderate to excellent yields (Scheme 3). The best yield was obtained for **5a** with 99%. Several functional groups, such as methyl, *N,N*-dimethylamino, and trifluoromethyl, are tolerated by the developed procedure. However, a fluorine group was converted to a hydroxy functional group (**5h**), most likely by nucleophilic aromatic substitution during aqueous workup. Interestingly, electron-donor groups, such as *N,N*-dimethylamino, proved to be beneficial in terms of yield when they are located at the alkyne-linked aryl group (Scheme 4a). In contrast, the *N,N*-dimethylanilino group is disadvantageous when located at position 5 of the uracil ring, which might be due to the protonation of the amine under the employed reaction conditions, making the aryl ring less feasible for the S_E_Ar reaction (Scheme 4b). The same effects may explain the cyclisation of **4f** to **5f**, while **4c** is not converted to the respective cyclisation product. Hence, the application of the reaction conditions to starting materials **4b**, **4c** and **4e** resulted in the decomposition of the starting materials and no product could be isolated. The employment of heterocyclic benzothiophene gave a very good yield of 80% for product **5i**.

### Optical properties

**Figure 3:**
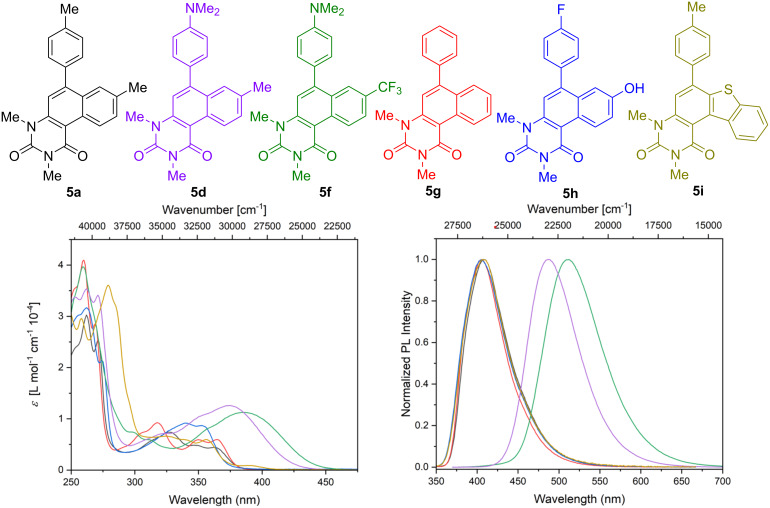
UV–vis absorption (left) and emission (right, λ_ex_ = 400 nm) spectra of **5a**, **5d**, **5f**, **5g**, **5h**, and **5i** in dichloromethane (*c* = 1⋅10^−5^ M).

All synthesized compounds exhibit photoluminescence by excitation with UV light. Hence, we studied the photophysical properties of all obtained derivatives **5** by steady-state absorption and fluorescence spectroscopy. The influence of the substitution pattern on the photophysical properties is displayed in Figure 3. The corresponding photophysical data and quantum yields are described in Table 2.

**Table 2:** Photophysical data of 1,3-dimethyl-5-phenyl-6-[2-(phenyl)ethynyl]uracil derivatives **5a**, **5d**, **5f**, **5g**, **5h**, and **5i** in dichloromethane (*c* = 1·10^−5^ M) at 20 °C.

	**5a**	**5d**	**5f**	**5g**	**5h**	**5i**

λ_1,abs_ (nm) ε_λ1_·10^4^ (M^−1^ cm^−1^)	262 3.1	253 3.4	258 4.0	260 4.1	262 3.2	258 3.0
λ_2,abs_ (nm) ε_λ2_·10^4^ (M^−1^ cm^−1^)	271 2.6	262 3.5	387 1.1	269 2.9	274 2.1	278 3.6
λ_3,abs_ (nm) ε_λ3_·10^4^ (M^−1^ cm^−1^)	327 0.8	271 3.4		317 0.9	339 0.9	356 0.6
λ _4,abs_ (nm) ε_λ4_·10^4^ (M^−1^ cm^−1^)	347 0.5	322 0.7		349 0.6	353 0.9	
λ _5,abs_ (nm) ε_λ5_·10^4^ (M^−1^ cm^−1^)	363 0.5	374 1.3		364 0.6		
λ_em_^335^ (nm)	408			406	405	408
λ_em_^355^ (nm)		487	512			
Φ^a^	12^b^	71^c^	51^c^	13^b^	8^b^	3^b^

^a^Fluorescence standard: quinine hemisulfate in H_2_SO_4_ (0.05 M) (Φ = 0.52) [70–71]; ^b^excitation at λ_ex_ = 335 nm; ^c^excitation at λ_ex_ = 355 nm.

The analysis of the absorption spectra revealed well-resolved bands for derivatives **5a**, **5d**, **5f**, **5g**, and **5i** for short wavelengths (250–300 nm). At higher wavelength (300–450 nm) broad absorption bands with a certain fine-structure for compounds **5a**, **5g**, and **5i** and structure-less absorption features for compounds **5d**, **5f**, and **5h** containing electron-donating functional groups are observed. In addition, the presence of strongly electron-donating *N*,*N*-dimethylamino groups leads to increased extinction coefficients and bathochromically shifted absorption bands which might be a result of a certain intramolecular charge transfer between the donating *N*,*N*-dimethylamino group and the electron-deficient uracil moiety. Similar effects are observed for the emission spectra. The emission maxima of *N*,*N*-dimethylamino-group-containing compounds **5d** and **5f** are bathochromically shifted by ≈80 nm and ≈100 nm, respectively. The other compounds show similar emission maxima at ≈405 nm, hinting to a limited impact of the aryl substituents and their functional groups on the emission properties for these compounds. Moreover, the presence of *N*,*N*-dimethylamino groups leads to strongly enhanced quantum yields up to 71%.

The highest quantum yield is observed for **5d** (71%), followed by **5f** with 51%. However, the presence of a pair of donor and acceptor groups appears to be disadvantageous in terms of quantum yield and the presence of only one donor group is advantageous. Interestingly, the quantum yields of **5a** (12%), **5g** (13%), **5h** (8%), and **5i** (3%) are comparatively lower than in case of **5g** and **5f**. It is reasonable to assume that this large difference is caused by the chosen substitution pattern and can be used for further investigation or modulation of desired properties.

## Conclusion

In summary, we have developed the synthesis of novel polycyclic uracil-based compounds. Careful optimisation of the reaction conditions led to the isolation of the desired products in excellent to moderate yields. The developed methodology tolerates various functional groups. The optical properties of as-prepared derivatives were investigated by steady-state absorption and photoluminescence spectroscopy. The photophysical properties are strongly altered by the presence *N*,*N*-dimethylaniline functional groups on the scaffold, which leads to strongly, bathochromically shifted absorption and emission spectra with elevated extinction coefficients and quantum yields up to 71%. Further studies will be directed to the synthesis to polycyclic, π-conjugated uracil derivatives.

## Experimental

### General information

Nuclear magnetic resonance spectra (^1^H/^13^C/^19^F NMR) were recorded on Bruker AVANCE 300 III, 250II, or 500 spectrometers. The analysed chemical shifts δ are referenced to the residual solvent signals of the deuterated solvents CDCl_3_ (δ = 7.26 ppm/77.16 ppm). Multiplicities due to spin–spin correlation are reported as follows: s = singlet, d = doublet, dd = double doublet, m = multiplet; they are further described by their coupling constants *J*. Infrared spectra (IR) were measured as attenuated total reflection (ATR) experiments using a Nicolet 380 FT-IR spectrometer. The signals were characterised by their wavenumbers and corresponding absorption as very strong (vs), strong (s), medium (m), weak (w) or very weak (vw). UV–vis spectra were recorded on a Cary 60 UV–vis spectrophotometer and emission spectra were recorded on an Agilent Cary Eclipse fluorescence spectrophotometer. Basic and high-resolution mass spectra (MS/HRMS) were measured on instruments coupled to a preceding gas chromatograph (GC) or liquid chromatograph (LC). Samples were ionised by electron impact ionisation (EI) on an Agilent 6890/5973 or Agilent 7890/5977 GC–MS equipped with an HP-5 capillary column using helium carrier gas or by electron spray ionisation (ESI) on an Agilent 1200/6210 Time-of-Flight (TOF) LC–MS. The solvent, toluene, was purchased as dry solvent and applied without further purification. Other reagents, catalysts, ligands, acids, and bases were used as purchased from commercial suppliers. Column chromatography was performed on Merck Silica gel 60 (particle size 63–200 μm). Solvents for extraction and column chromatography were distilled prior employment.

### Synthesis of **4a**–**i**

The synthesis of **4a**–**f** has been previously reported. Novel derivatives **4g**–**i** were prepared according to our previously reported procedure [65].

**1,3-Dimethyl-5-phenyl-6-(phenylethynyl)pyrimidine-2,4(1*H*,3*H*)-dione (4g).** Compound **4g** was obtained as a brown solid in 58% yield (58.3 mg, 184 µmol, *R*_f_ 0.19 (heptane/ethyl acetate 3:2)); mp 152–154 °C; IR (ATR) ν̃: 1695 (s), 1642 (vs), 1582 (s), 1493 (s), 1440 (s), 1421 (s), 1176 (m), 1079 (m), 756 (s) cm^−1^; ^1^H NMR (500 MHz, chloroform-*d*) δ 7.51–7.48 (m, 2H), 7.45–7.41 (m, 2H), 7.41–7.36 (m, 2H), 7.34–7.29 (m, 2H), 7.24–7.21 (m, 2H), 3.71 (s, 3H), 3.45 (s, 3H); ^13^C {^1^H} NMR (126 MHz, chloroform-*d*) δ 162.1, 151.6, 134.3, 133.2, 131.9, 130.9, 130.5, 128.7, 128.3, 128.0, 120.7, 119.0, 104.2, 81.0, 34.6, 28.7; EIMS (70 eV) *m*/*z* (%): 315 (100, M^+^), 258 (26), 230 (67), 215 (17), 202 (23), 189 (13); HRESIMS-TOF (*m*/*z*): [M + H]^+^ calcd for C_20_H_17_N_2_O_2_, 317.1290; found, 317.1282.

**5-(4-Fluorophenyl)-6-((4-fluorophenyl)ethynyl)-1,3-dimethylpyrimidine-2,4(1*H*,3*H*)-dione (4h).** Compound **4h** was obtained as a brown solid in 43% yield (46.2 mg, 131 µmol, *R*_f_ 0.17 (heptane/ethyl acetate 3:2)); mp 187–189 °C; IR (ATR) ν̃: 1708 (s), 1654 (vs), 1574 (s), 1514 (s), 1506 (s), 1446 (s), 1423 (s), 1232 (s), 1158 (s) cm^−1^; ^1^H NMR (500 MHz, chloroform-*d*) δ 7.48–7.44 (m, 2H), 7.26–7.22 (m, 2H), 7.15–7.10 (m, 2H), 7.06–7.02 (m, 2H), 3.70 (s, 3H), 3.44 (s, 3H); ^19^F NMR (471 MHz, chloroform-*d*) δ −113.3, −106.3; ^13^C {^1^H} NMR (126 MHz, chloroform-*d*) δ 163.9 (d, *J* = 253.7 Hz), 162.7 (d, *J* = 247.8 Hz), 162.0, 151.5, 134.3, 134.1 (d, *J* = 9.0 Hz), 132.8 (d, *J* = 8.2 Hz), 129.1 (d, *J* = 3.3 Hz), 117.9, 116.6 (d, *J* = 3.6 Hz), 116.4 (d, *J* = 22.4 Hz), 115.1 (d, *J* = 21.6 Hz), 103.3, 80.7, 34.7, 28.7; EIMS (70 eV) *m*/*z* (%): 352 (96, M^+^), 294 (28), 266 (67), 251 (14), 238 (30), 160 (100); HRESIMS-TOF(*m*/*z*): [M + H]^+^ calcd for C_20_H_15_F_2_N_2_O_2_, 353.1101; found, 353.1100.

**5-(Benzo[*b*]thiophen-3-yl)-1,3-dimethyl-6-(*p*-tolylethynyl)pyrimidine-2,4(1*H*,3*H*)-dione (4i).** Compound **4i** was obtained as a brownish solid in 54% yield (189 mg, 488 µmol, *R*_f_ 0.21 (heptane/ethyl acetate 3:2)); mp 162–164 °C; IR (ATR) ν̃: 1695 (s), 1640 (vs), 1582 (s), 1510 (s), 1446 (s), 1428 (s), 1219 (m), 813 (s) cm^−1^; ^1^H NMR (300 MHz, chloroform-*d*) δ 7.94–7.87 (m, 1H), 7.65–7.59 (m, 1H), 7.58 (s, 1H), 7.39–7.31 (m, 2H), 7.06–7.01 (m, 2H), 6.86–6.80 (m, 2H), 3.73 (s, 3H), 3.48 (s, 3H), 2.30 (s, 3H); ^13^C {^1^H} NMR (75 MHz, chloroform-*d*) δ 161.7, 151.7, 141.2, 139.7, 138.3, 135.9, 131.9, 129.4, 128.5, 128.2, 124.3, 124.2, 123.4, 122.8, 117.2, 112.6, 106.2, 80.4, 34.7, 28.7, 21; EIMS (70 eV) *m*/*z* (%): 386 (58, M^+^), 371 (100), 300 (68), 286 (51), 271 (18), 268 (26); HRESIMS-TOF (*m*/*z*): [M + H]^+^ calcd for C_23_H_19_N_2_O_2_S, 387.1167; found, 387.1171.

### General procedure for the preparation of **5**

A mixture of the corresponding starting material **4** (0.145 mmol) and *p*-TsOH·H_2_O (20 equiv; 2.94 mmol; 559 mg) was dissolved in dry toluene (2 mL) and stirred for 4 hours under argon atmosphere at 100 °C. The reaction was monitored by TLC until completion. The reaction was neutralised with a saturated NaHCO_3_ solution and diluted with water (40 mL). The phases were separated, and the aqueous layer was extracted with dichloromethane (3 × 30 mL). The combined organic layers were dried over Na_2_SO_4_, concentrated under reduced pressure, and purified by column chromatography (heptane/ethyl acetate).

**2,4,8-Trimethyl-6-(*p*-tolyl)benzo[*f*]quinazoline-1,3(2*H*,4H)-dione (5a).** According to general procedure, compound **5a** was obtained as a brown solid in 99% yield (49.3 mg, 143 µmol, *R*_f_ 0.29 (heptane/ethyl acetate 3:2)); mp 185–187 °C; IR (ATR) ν̃: 1685 (s), 1640 (vs), 1508 (s), 1421 (s), 1306 (m), 1121 (m), 1022 (m), 820 (s), 748 (s) cm^−1^; ^1^H NMR (500 MHz, chloroform-*d*) δ 9.82–9.78 (m, 1H), 7.76 (d, *J* = 8.5 Hz, 1H), 7.39–7.34 (m, 4H), 7.30 (dd, *J* = 8.6, 1.8 Hz, 1H), 7.27 (s, 1H), 3.72 (s, 3H), 3.59 (s, 3H), 2.60 (d, *J* = 0.8 Hz, 3H), 2.48 (s, 3H); ^13^C {^1^H} NMR (126 MHz, chloroform-*d*) δ 162.7, 151.5, 149.0, 141.4, 140.0, 138.5, 136.9, 132.6, 129.7, 129.3, 127.7, 126.8, 126.7, 125.5, 113.5, 106.4, 31.8, 28.8, 22.6, 21.4; EIMS (70 eV) *m*/*z* (%): 344 (M^+^, 100), 258 (9), 232 (9), 215 (10), 202 (8); HREIMS (*m*/*z*): [M]^+^ calcd for C_22_H_20_N_2_O_2_, 344.15173; found, 344.15193.

**6-(4-(*N*,*N*-Dimethylamino)phenyl)-2,4,8-trimethylbenzo[*f*]quinazoline-1,3(2*H*,4*H*)-dione (5d).** According to general procedure, compound **5d** was obtained as a brown solid in 92% yield (70 mg, 187 µmol, *R*_f_ 0.24 (heptane/ethyl acetate 3:2)); mp: 207–209 °C; IR (ATR) ν̃: 1697 (vs), 1642 (vs), 1512 (s), 1423 (s), 1201 (s), 1129 (s), 1036 (m), 820 (vs), 745 (s) cm^−1^; ^1^H NMR (300 MHz, chloroform-*d*) δ 9.81–9.74 (m, 1H), 7.90 (d, *J* = 8.6 Hz, 1H), 7.44–7.33 (m, 2H), 7.30 (dd, *J* = 8.5, 1.7 Hz, 1H), 7.24 (s, 1H), 6.92–6.80 (m, 2H), 3.70 (s, 3H), 3.57 (s, 3H), 3.06 (s, 6H), 2.59 (s, 3H); ^13^C {^1^H} NMR (75 MHz, chloroform-*d*) δ 162.7, 151.5, 150.6, 149.3, 141.5, 139.7, 132.7, 130.8, 127.4, 127.3, 127.0, 126.8, 125.5, 113.2, 112.1, 105.7, 40.6, 31.8, 28.8, 22.5; EIMS (70 eV) *m*/*z* (%): 373 (M^+^, 100), 246 (22), 281 (7), 207 (15), 202 (8); HRESIMS-TOF (*m*/*z*): [M + H]^+^ calcd for C_23_H_24_N_3_O_2_, 374.1868; found, 374.1859.

**6-(4-(*N*,*N*-Dimethylamino)phenyl)-2,4-dimethyl-8-(trifluoromethyl)benzo[*f*]quinazoline-1,3-(2*H*,4*H*)-dione (5f).** According to general procedure, compound **5f** was obtained as a brown solid in 70% yield (45.8 mg, 107 µmol, *R*_f_ 0.21 (heptane/ethyl acetate 3:2)); mp 259–261 °C; IR (ATR) ν̃: 1691 (s), 1596 (s), 1473 (s), 1314 (vs), 1147 (s), 1116 (vs), 1075 (vs), 818 (vs), 754 (m) cm^−1^; ^1^H NMR (300 MHz, chloroform-*d*) δ 10.10 (d, *J* = 9.2 Hz, 1H), 8.32 (s, 1H), 7.86 (dd, *J* = 9.2, 2.1 Hz, 1H), 7.44–7.35 (m, 3H), 6.92–6.84 (m, 2H), 3.75 (s, 3H), 3.59 (s, 3H), 3.09 (s, 6H); ^19^F NMR (282 MHz, chloroform-*d*) δ −62.2; ^13^C {^1^H} NMR (75 MHz, chloroform-*d*) δ 162.3, 151.4, 150.9, 150.2, 142.6, 134.4, 130.8, 127.7, 127.3, 127.1 (q, *J* = 32.6 Hz), 126.0, 124.9 (q, *J* = 2.8 Hz), 124.7 (q, *J* = 4.7 Hz), 124.4 (q, *J* = 272.1 Hz), 115.3, 112.3, 106.0, 40.5, 31.9, 28.9; EIMS (70 eV) *m*/*z* (%): 427 (M^+^, 100), 369 (5), 270 (4); HRESIMS-TOF (*m*/*z*): [M + H]^+^ calcd for C_23_H_21_F_3_N_3_O_2_, 428.1586; found, 428.1577.

**2,4-Dimethyl-6-phenylbenzo[*f*]quinazoline-1,3(2*H*,4*H*)-dione (5g).** According to general procedure, compound **5g** was obtained as a brown solid in 44% yield (22.2 mg, 70.2 µmol, *R*_f_ 0.26 (heptane/ethyl acetate 3:2)); mp 193–195 °C; IR (ATR) ν̃: 1693 (s), 1572 (s), 1423 (s), 1341 (s), 1125 (m), 1030 (m), 853 (m), 776 (vs), 766 (s) cm^−1^; ^1^H NMR (500 MHz, chloroform-*d*) δ 10.0–9.9 (m, 1H), 7.8 (dd, *J* = 8.4, 1.4 Hz, 1H), 7.7 (ddd, *J* = 8.6, 6.8, 1.5 Hz, 1H), 7.6–7.5 (m, 3H), 7.5–7.4 (m, 3H), 7.4 (s, 1H), 3.7 (s, 3H), 3.6 (s, 3H); ^13^C {^1^H} NMR (126 MHz, chloroform-*d*) δ 162.5, 151.4, 149.0, 141.2, 139.7, 132.3, 129.8, 129.7, 128.7, 128.6, 128.4, 126.9, 126.2, 125.7, 114.6, 107.0, 31.8, 28.9 (signals of two carbons are absent, which may relate to signal overlap); EIMS (70 eV) *m*/*z* (%): 316 (M^+^, 100), 259 (16), 230 (16), 202 (9), 189 (5); HREIMS (*m*/*z*): [M]^+^ calcd for C_20_H_16_N_2_O_2_, 316.12063; found, 316.12044.

**6-(4-Fluorophenyl)-8-hydroxy-2,4-dimethylbenzo[*f*]quinazoline-1,3(2*H*,4*H*)-dione (5h).** According to general procedure, compound **5h** was obtained as a brown solid in 45% yield (21.7 mg, 61.9 µmol, *R*_f_ 0.13 (heptane/ethyl acetate 3:2)); mp 317–319 °C; IR (ATR) ν̃: 1607 (vs), 1506 (s), 1423 (s), 1355 (s), 1230 (vs), 1224 (vs), 1090 (m), 832 (s), 748 (s) cm^−1^; ^1^H NMR (300 MHz, chloroform-*d*) δ 10.2 (s, 1H), 9.3 (d, *J* = 2.5 Hz, 1H), 7.6–7.5 (m, 3H), 7.4–7.4 (m, 2H), 7.3 (s, 1H), 7.1 (dd, *J* = 9.1, 2.6 Hz, 1H), 3.6 (s, 3H), 3.4 (s, 3H); ^19^F NMR (282 MHz, chloroform-*d*) δ −113.9; ^13^C {^1^H} NMR (75 MHz, chloroform-*d*) δ 162.6 (d, *J* = 245.3 Hz), 162.3, 159.1, 151.0, 146.9, 142.2, 136.1 (d, *J* = 3.3 Hz), 134.1, 132.2 (d, *J* = 8.3 Hz), 128.5, 122.2, 117.7, 115.9 (d, *J* = 21.4 Hz), 112.6, 108.8, 105.0, 31.9, 28.8; EIMS (70 eV) *m*/*z* (%): 350 (M^+^, 100), 295 (14), 267 (16), 164 (13), 238 (23), 160 (21); HREIMS (*m*/*z*): [M]^+^ calcd for C_20_H_15_FN_2_O_3_, 350.1062; found, 350.10584.

**2,4-Dimethyl-6-(*p*-tolyl)benzo[4,5]thieno[3,2-*f*]quinazoline-1,3(2*H*,4*H*)-dione (5i).** According to general procedure, compound **5i** was obtained as a brown solid in 80% yield (60.1 mg, 155 µmol, *R*_f_ 0.22 (heptane/ethyl acetate 3:2)); mp 255–257 °C; IR (ATR) ν̃: 1648 (vs), 1483 (s), 1419 (s), 1333 (m), 1158 (m), 1030 (m), 914 (m), 820 (s), 743 (vs) cm^−1^; ^1^H NMR (300 MHz, chloroform-*d*) δ 7.89 (d, *J* = 7.9 Hz, 1H), 7.39–7.34 (m, 5H), 7.15–7.08 (m, 2H), 7.05 (s, 1H), 3.64 (s, 3H), 3.56 (s, 3H), 2.53 (s, 3H); ^13^C {^1^H} NMR (75 MHz, chloroform-*d*) δ 161.8, 151.2, 145.7, 141.7, 141.7, 139.2, 138.7, 137.3, 133.8, 129.7, 129.5, 128.6, 126.1, 124.2, 124.1, 122.6, 113.0, 108.3, 31.5, 28.7, 21.6; EIMS (70 eV) *m*/*z* (%): 386 (M^+^, 100), 300 (13), 258 (14); HRESIMS-TOF (*m*/*z*): [M + H]^+^ calcd for C_23_H_19_N_2_O_2_S, 387.1167; found, 387.1169.

## Supporting Information

File 1 Copies of NMR spectra.

## Data Availability

All data that supports the findings of this study is available in the published article and/or the supporting information to this article.
