# Supplementary material for: Synthesis of benzo[f]quinazoline-1,3(2H,4H)-diones
Source: Beilstein J Org Chem. 2024 Oct 28;20:2708–19. doi: 10.3762/bjoc.20.228 (PMC11533120; doi:10.3762/bjoc.20.228)

BEILSTEIN JOURNAL OF ORGANIC CHEMISTRY

# Supporting Information

for

# Synthesis of benzo[*f*]quinazoline-1,3(2*H*,4*H*)-diones

Ruben Manuel Figueira de Abreu, Peter Ehlers and Peter Langer

*Beilstein J. Org. Chem.* **2024,** *20,* 2708–2719. doi:10.3762/bjoc.20.228

# Copies of NMR spectra

License and Terms: This is a supporting information file under the terms of the Creative Commons Attribution License (https://creativecommons.org/licenses/by/4.0). Please note that the reuse, redistribution and reproduction in particular requires that the author(s) and source are credited and that individual graphics may be subject to special legal provisions.
The license is subject to the *Beilstein Journal of Organic Chemistry* terms and conditions: (https://www.beilstein-journals.org/bjoc/terms)

# $^1$H, $^{13}$C, and $^{19}$F NMR spectra

## 1,3-Dimethyl-5-phenyl-6-(phenylethynyl)pyrimidine-2,4(1*H*,3*H*)-dione (4g).

| | Parameter | Value |
|---|---|---|
| 1 | Solvent | CDCl3 |
| 2 | Temperature | 298.1 |
| 3 | Pulse Sequence | zg30 |
| 4 | Experiment | 1D |
| 5 | Number of Scans | 16 |
| 6 | Receiver Gain | 101.0 |
| 7 | Relaxation Delay | 1.0000 |
| 8 | Pulse Width | 8.0000 |
| 9 | Spectrometer Frequency | 500.13 |
| 10 | Spectral Width | 10000.0 |
| 11 | Lowest Frequency | -1924.0 |
| 12 | Nucleus | 1H |
| 13 | Acquired Size | 32768 |
| 14 | Spectral Size | 65536 |

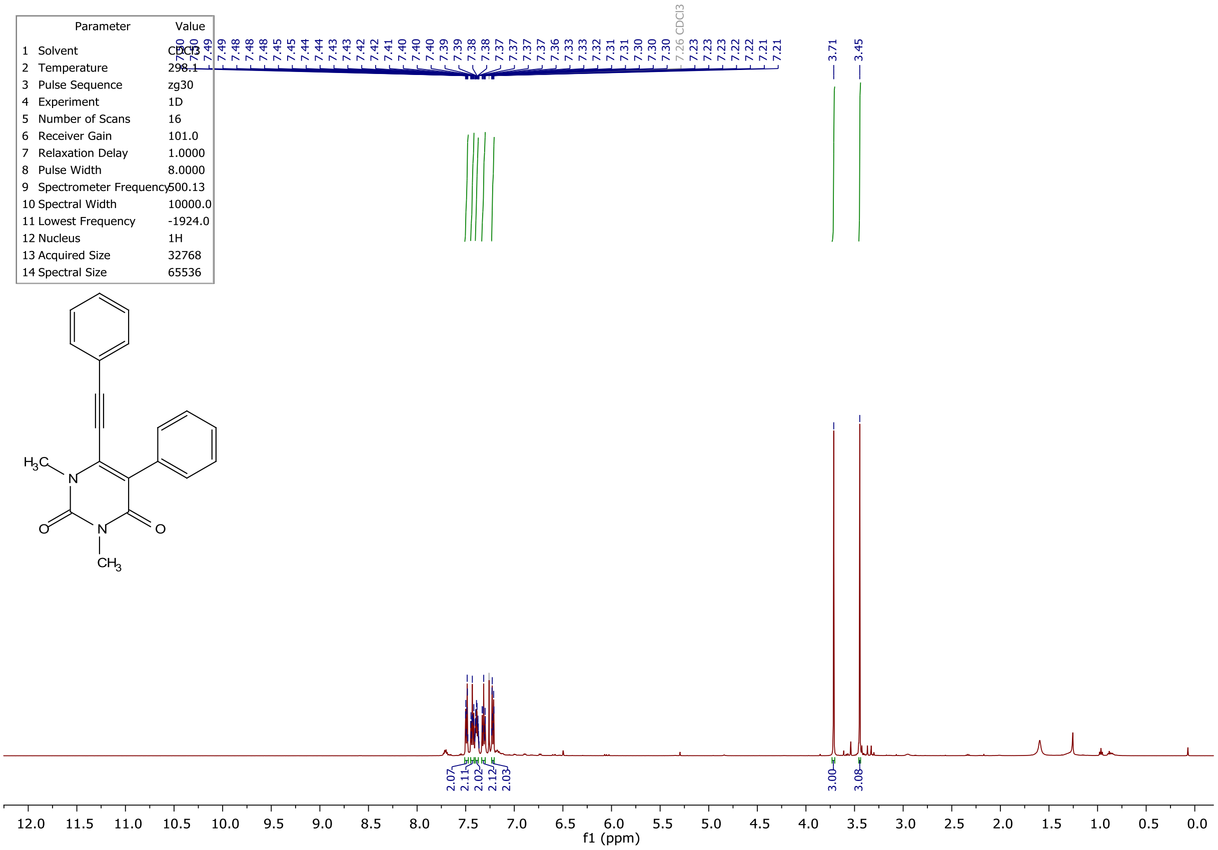


| | Parameter | Value |
|---|---|---|
| 1 | Solvent | CDCl3 |
| 2 | Temperature | 298.1 |
| 3 | Pulse Sequence | zgpg30 |
| 4 | Experiment | 1D |
| 5 | Number of Scans | 1024 |
| 6 | Receiver Gain | 101.0 |
| 7 | Relaxation Delay | 2.0000 |
| 8 | Pulse Width | 10.0000 |
| 9 | Spectrometer Frequency | 125.77 |
| 10 | Spectral Width | 30120.5 |
| 11 | Lowest Frequency | -2469.0 |
| 12 | Nucleus | 13C |
| 13 | Acquired Size | 32768 |
| 14 | Spectral Size | 65536 |

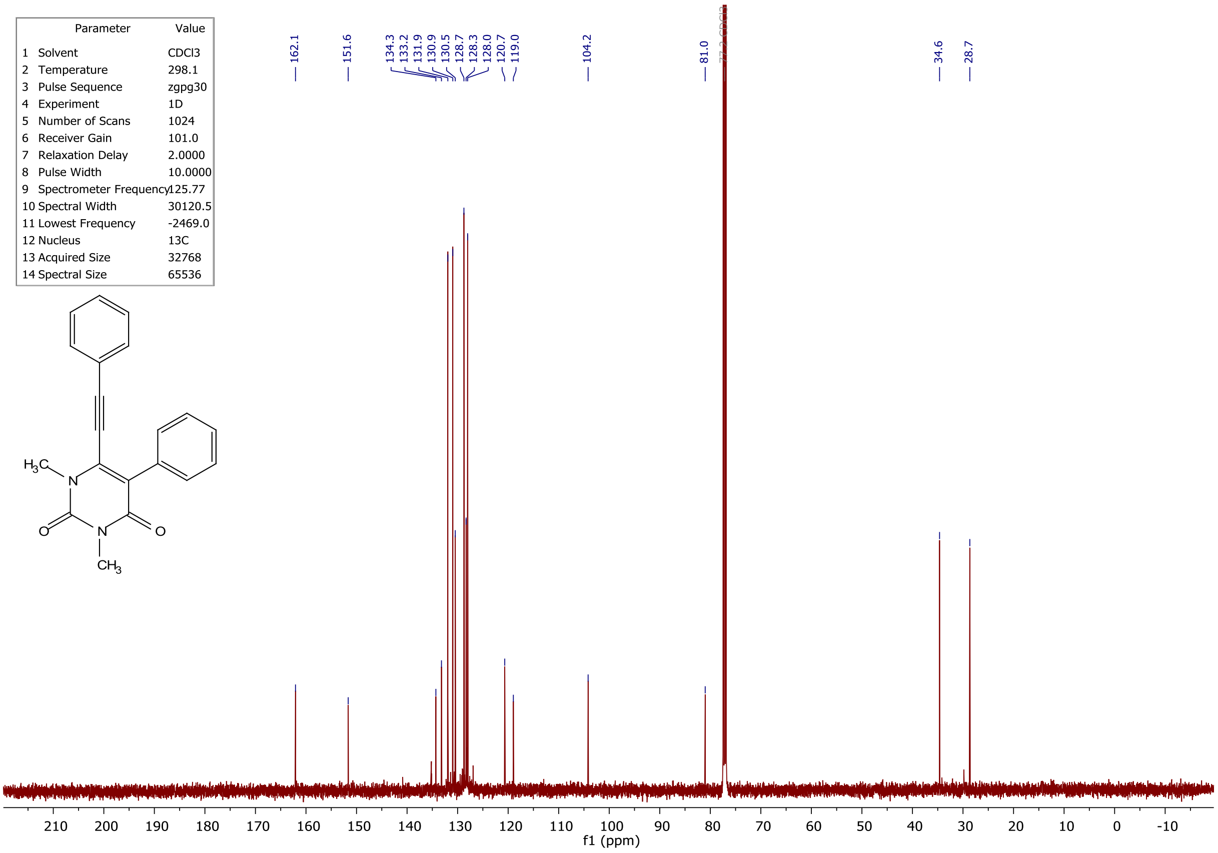

## 5-(4-Fluorophenyl)-6-((4-fluorophenyl)ethynyl)-1,3-dimethylpyrimidine-2,4(1*H*,3*H*)-dione (4h)

| | Parameter | Value |
|---|---|---|
| 1 | Solvent | CDCl3 |
| 2 | Temperature | 298.1 |
| 3 | Pulse Sequence | zg30 |
| 4 | Experiment | 1D |
| 5 | Number of Scans | 16 |
| 6 | Receiver Gain | 101.0 |
| 7 | Relaxation Delay | 1.0000 |
| 8 | Pulse Width | 8.0000 |
| 9 | Spectrometer Frequency | 500.13 |
| 10 | Spectral Width | 10000.0 |
| 11 | Lowest Frequency | -1924.0 |
| 12 | Nucleus | 1H |
| 13 | Acquired Size | 32768 |
| 14 | Spectral Size | 65536 |

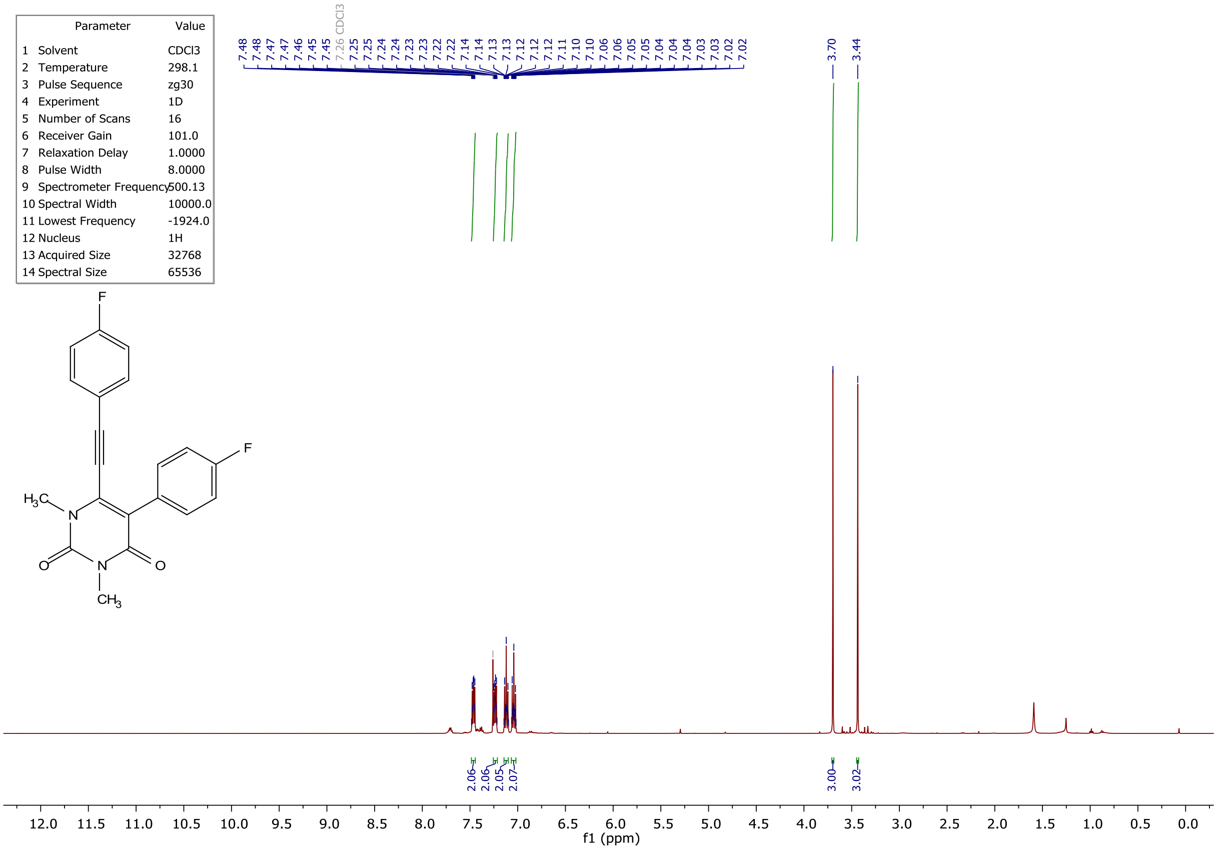


| | Parameter | Value |
|---|---|---|
| 1 | Solvent | CDCl3 |
| 2 | Temperature | 298.3 |
| 3 | Pulse Sequence | zgig |
| 4 | Experiment | 1D |
| 5 | Number of Scans | 16 |
| 6 | Receiver Gain | 101.0 |
| 7 | Relaxation Delay | 1.0000 |
| 8 | Pulse Width | 12.0000 |
| 9 | Spectrometer Frequency | 470.55 |
| 10 | Spectral Width | 113636.4 |
| 11 | Lowest Frequency | -103877.4 |
| 12 | Nucleus | 19F |
| 13 | Acquired Size | 65536 |
| 14 | Spectral Size | 131072 |

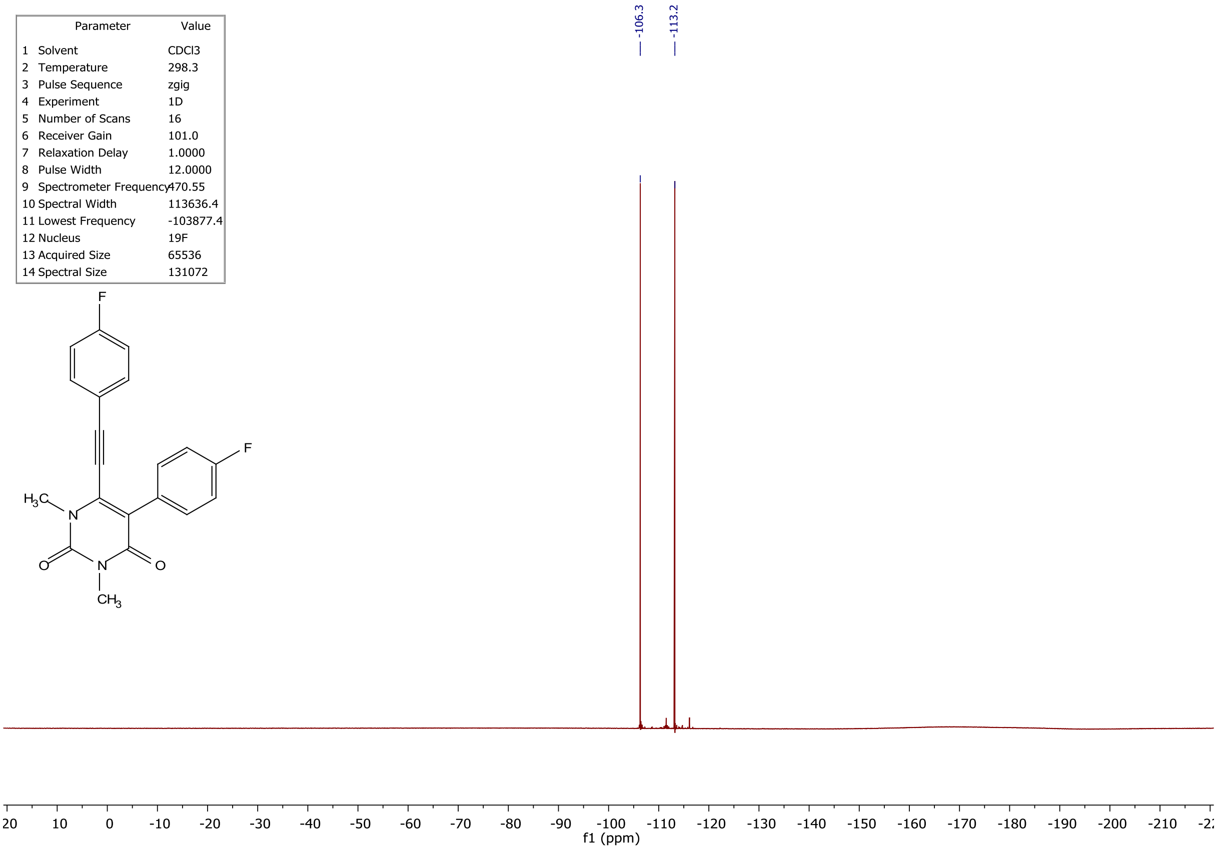

| | Parameter | Value |
|---|---|---|
| 1 | Solvent | CDCl3 |
| 2 | Temperature | 298.2 |
| 3 | Pulse Sequence | zgpg30 |
| 4 | Experiment | 1D |
| 5 | Number of Scans | 2048 |
| 6 | Receiver Gain | 101.0 |
| 7 | Relaxation Delay | 2.0000 |
| 8 | Pulse Width | 10.0000 |
| 9 | Spectrometer Frequency | 125.77 |
| 10 | Spectral Width | 30120.5 |
| 11 | Lowest Frequency | -2468.6 |
| 12 | Nucleus | 13C |
| 13 | Acquired Size | 32768 |
| 14 | Spectral Size | 65536 |

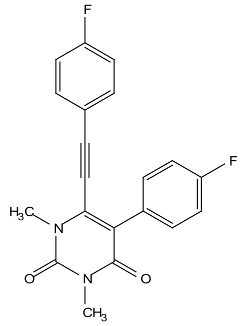


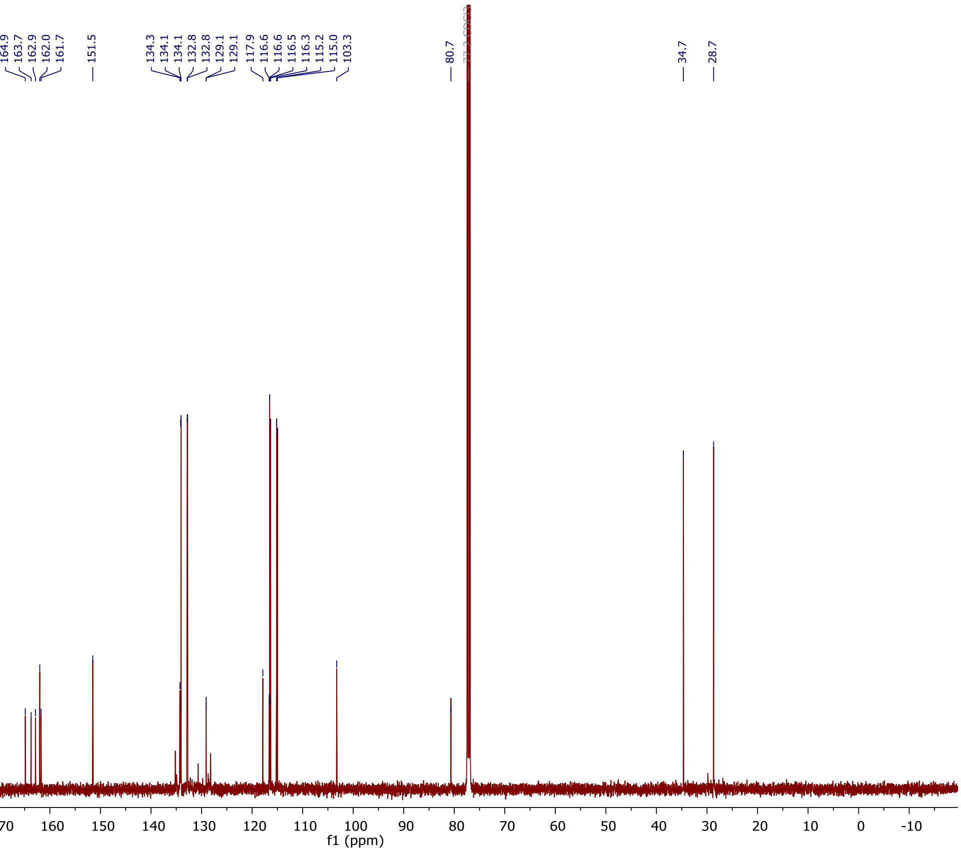

# 5-(Benzo[*b*]thiophen-3-yl)-1,3-dimethyl-6-(*p*-tolylethynyl)pyrimidine-2,4(1*H*,3*H*)-dione (4i)

| | Parameter | Value |
|---|---|---|
| 1 | Solvent | CDCl3 |
| 2 | Temperature | 298.2 |
| 3 | Pulse Sequence | zg30 |
| 4 | Experiment | 1D |
| 5 | Number of Scans | 16 |
| 6 | Receiver Gain | 228.0 |
| 7 | Relaxation Delay | 2.0000 |
| 8 | Pulse Width | 10.0000 |
| 9 | Spectrometer Frequency | 300.13 |
| 10 | Spectral Width | 6188.1 |
| 11 | Lowest Frequency | -1246.9 |
| 12 | Nucleus | 1H |
| 13 | Acquired Size | 32768 |
| 14 | Spectral Size | 65536 |

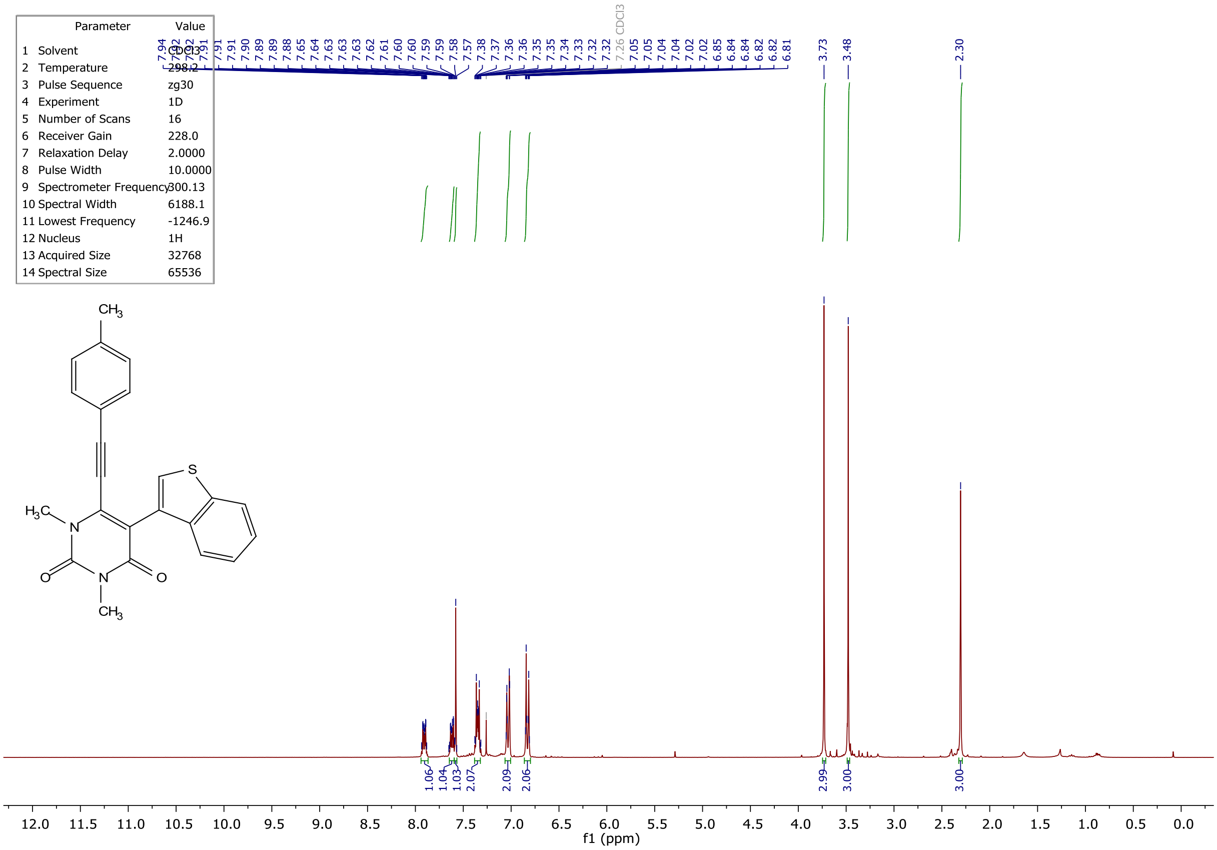


| | Parameter | Value |
|---|---|---|
| 1 | Solvent | CDCl3 |
| 2 | Temperature | 298.2 |
| 3 | Pulse Sequence | zgpg30 |
| 4 | Experiment | 1D |
| 5 | Number of Scans | 1024 |
| 6 | Receiver Gain | 2050.0 |
| 7 | Relaxation Delay | 2.0000 |
| 8 | Pulse Width | 9.6000 |
| 9 | Spectrometer Frequency | 75.48 |
| 10 | Spectral Width | 18028.8 |
| 11 | Lowest Frequency | -1460.8 |
| 12 | Nucleus | 13C |
| 13 | Acquired Size | 32768 |
| 14 | Spectral Size | 65536 |

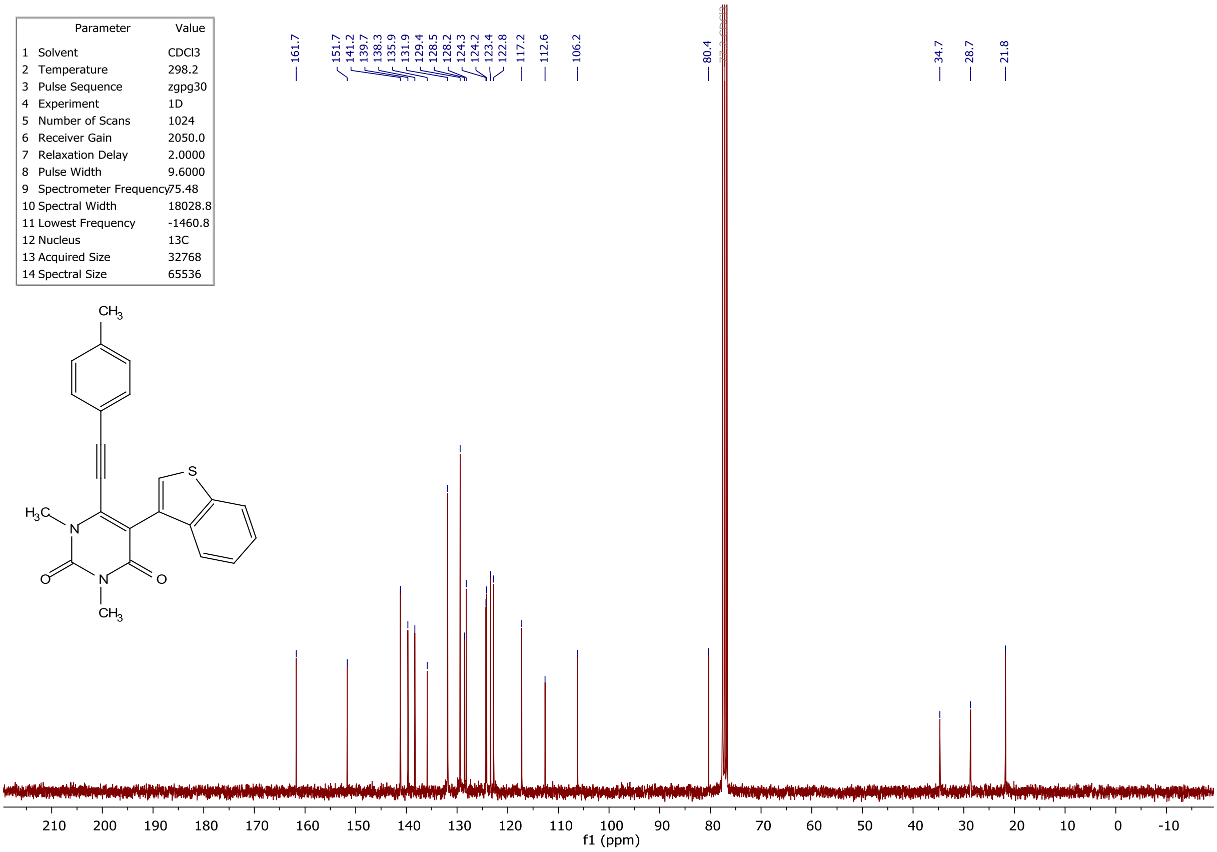

## 2,4,8-Trimethl-6-(*p*-tolyl)benzo[*f*]quinazoline-1,3(2*H*,4H)-dione (5a)

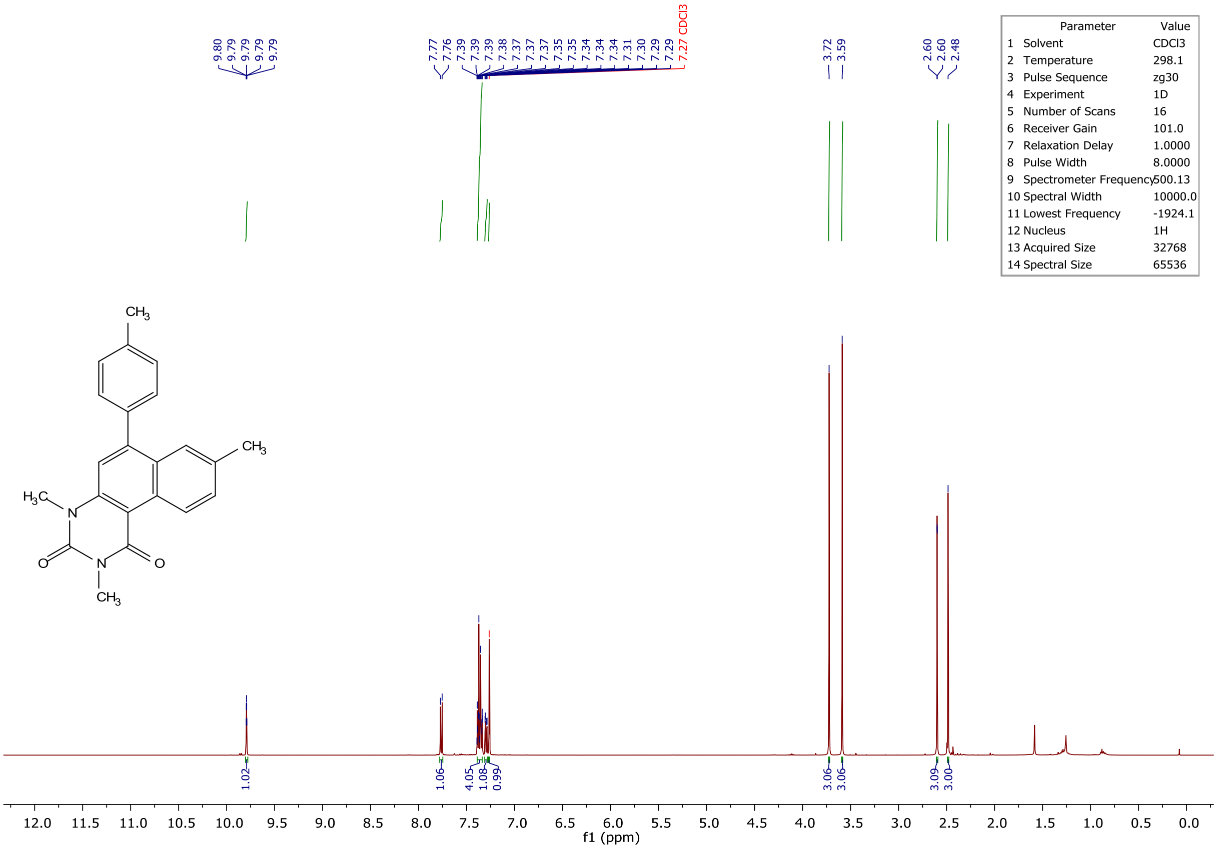


| | Parameter | Value |
|---|---|---|
| 1 | Solvent | CDCl3 |
| 2 | Temperature | 298.1 |
| 3 | Pulse Sequence | zg30 |
| 4 | Experiment | 1D |
| 5 | Number of Scans | 16 |
| 6 | Receiver Gain | 101.0 |
| 7 | Relaxation Delay | 1.0000 |
| 8 | Pulse Width | 8.0000 |
| 9 | Spectrometer Frequency | 500.13 |
| 10 | Spectral Width | 10000.0 |
| 11 | Lowest Frequency | -1924.1 |
| 12 | Nucleus | 1H |
| 13 | Acquired Size | 32768 |
| 14 | Spectral Size | 65536 |

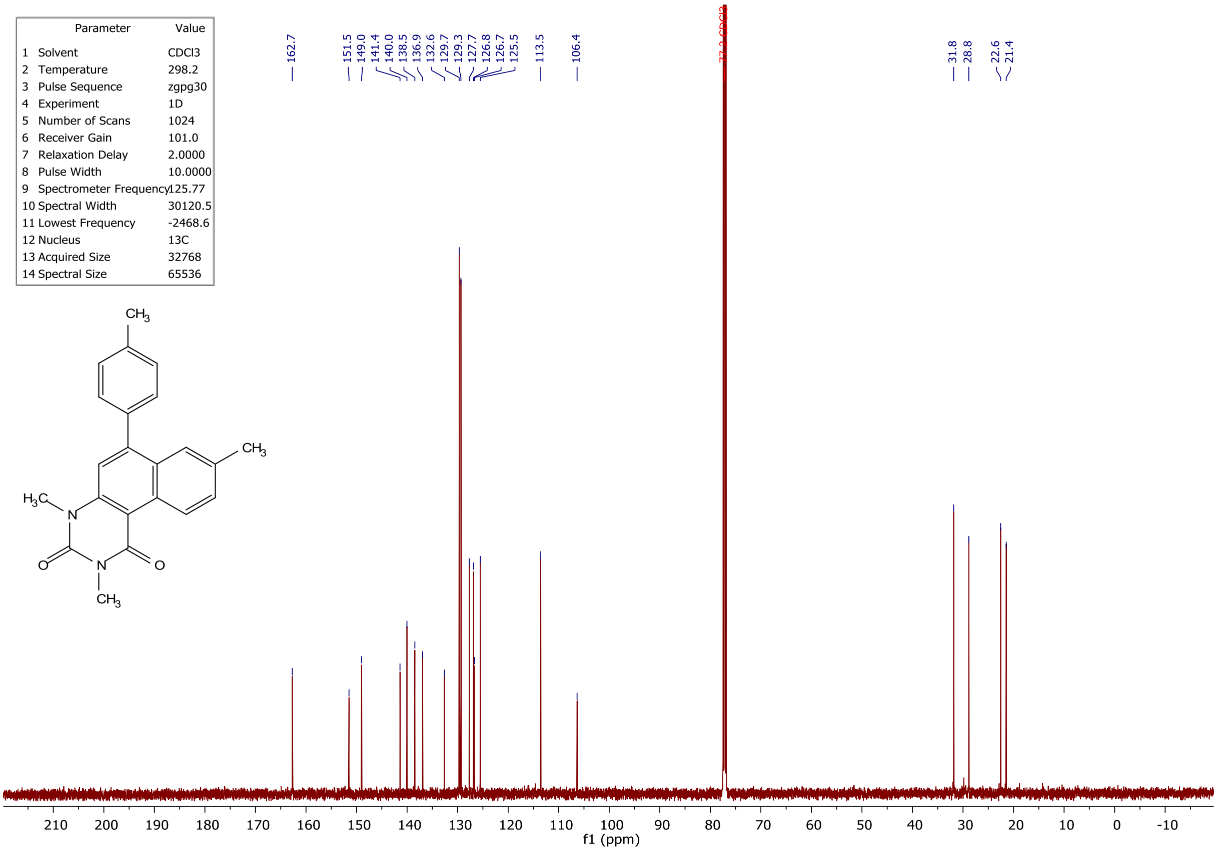


| | Parameter | Value |
|---|---|---|
| 1 | Solvent | CDCl3 |
| 2 | Temperature | 298.2 |
| 3 | Pulse Sequence | zgpg30 |
| 4 | Experiment | 1D |
| 5 | Number of Scans | 1024 |
| 6 | Receiver Gain | 101.0 |
| 7 | Relaxation Delay | 2.0000 |
| 8 | Pulse Width | 10.0000 |
| 9 | Spectrometer Frequency | 125.77 |
| 10 | Spectral Width | 30120.5 |
| 11 | Lowest Frequency | -2468.6 |
| 12 | Nucleus | 13C |
| 13 | Acquired Size | 32768 |
| 14 | Spectral Size | 65536 |

# 6-(4-(*N*,*N*-Dimethylamino)phenyl)-2,4,8-trimethylbenzo[*f*]quinazoline-1,3(2*H*,4*H*)-dione (5d)

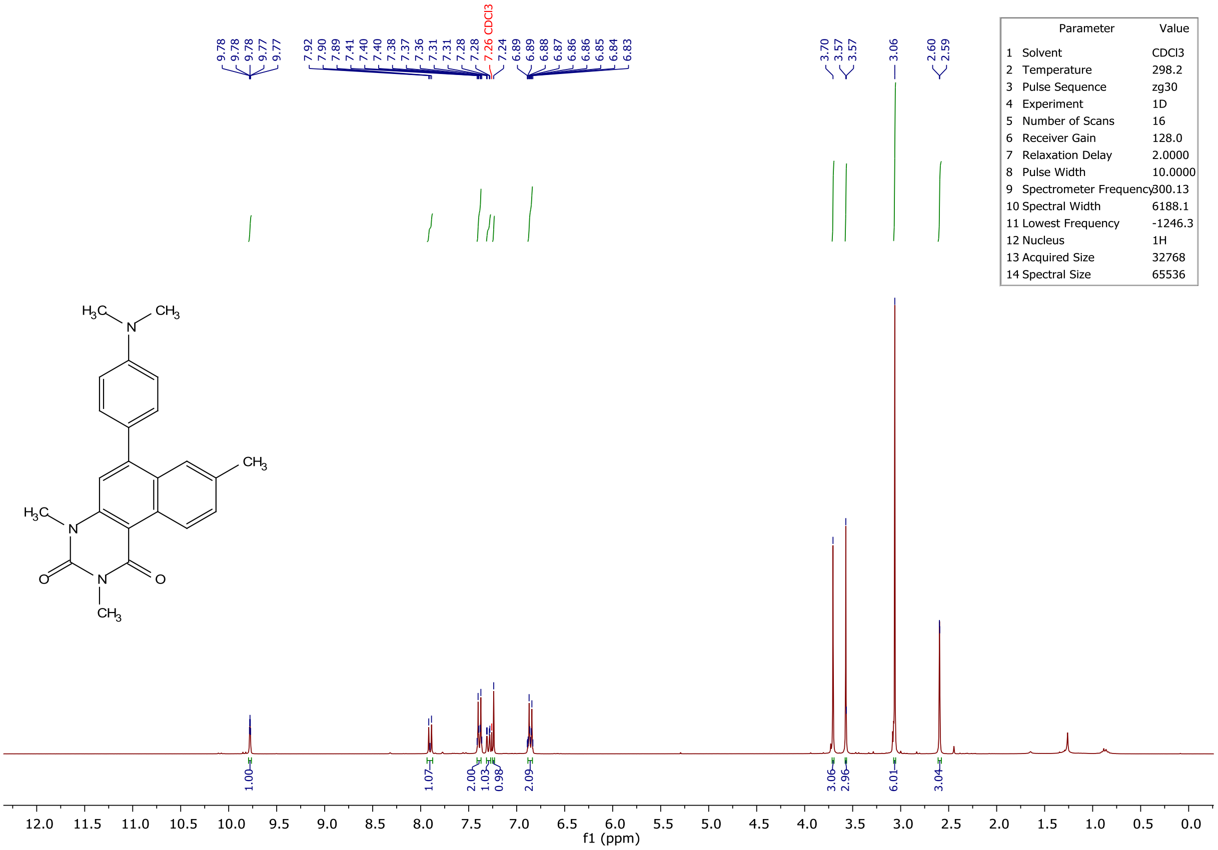


| Parameter | Value |
|---|---|
| 1 Solvent | CDCl3 |
| 2 Temperature | 298.2 |
| 3 Pulse Sequence | zg30 |
| 4 Experiment | 1D |
| 5 Number of Scans | 16 |
| 6 Receiver Gain | 128.0 |
| 7 Relaxation Delay | 2.0000 |
| 8 Pulse Width | 10.0000 |
| 9 Spectrometer Frequency | 300.13 |
| 10 Spectral Width | 6188.1 |
| 11 Lowest Frequency | -1246.3 |
| 12 Nucleus | 1H |
| 13 Acquired Size | 32768 |
| 14 Spectral Size | 65536 |

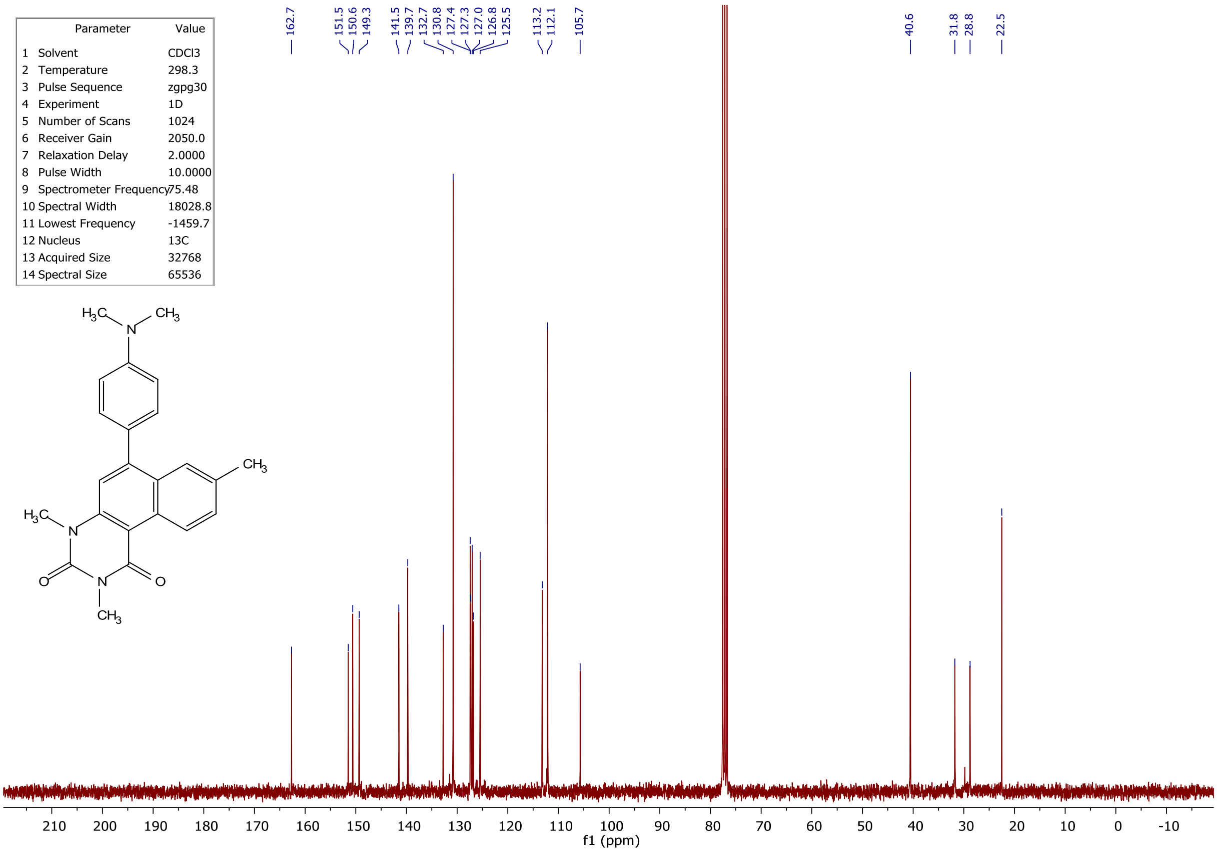


| Parameter | Value |
|---|---|
| 1 Solvent | CDCl3 |
| 2 Temperature | 298.3 |
| 3 Pulse Sequence | zgpg30 |
| 4 Experiment | 1D |
| 5 Number of Scans | 1024 |
| 6 Receiver Gain | 2050.0 |
| 7 Relaxation Delay | 2.0000 |
| 8 Pulse Width | 10.0000 |
| 9 Spectrometer Frequency | 75.48 |
| 10 Spectral Width | 18028.8 |
| 11 Lowest Frequency | -1459.7 |
| 12 Nucleus | 13C |
| 13 Acquired Size | 32768 |
| 14 Spectral Size | 65536 |

**6-(4-(*N*,*N*-Dimethylamino)phenyl)-2,4-dimethyl-8-(trifluoromethyl)benzo[*f*]quinazoline-1,3-(2*H*,4*H*)-dione (5f)**

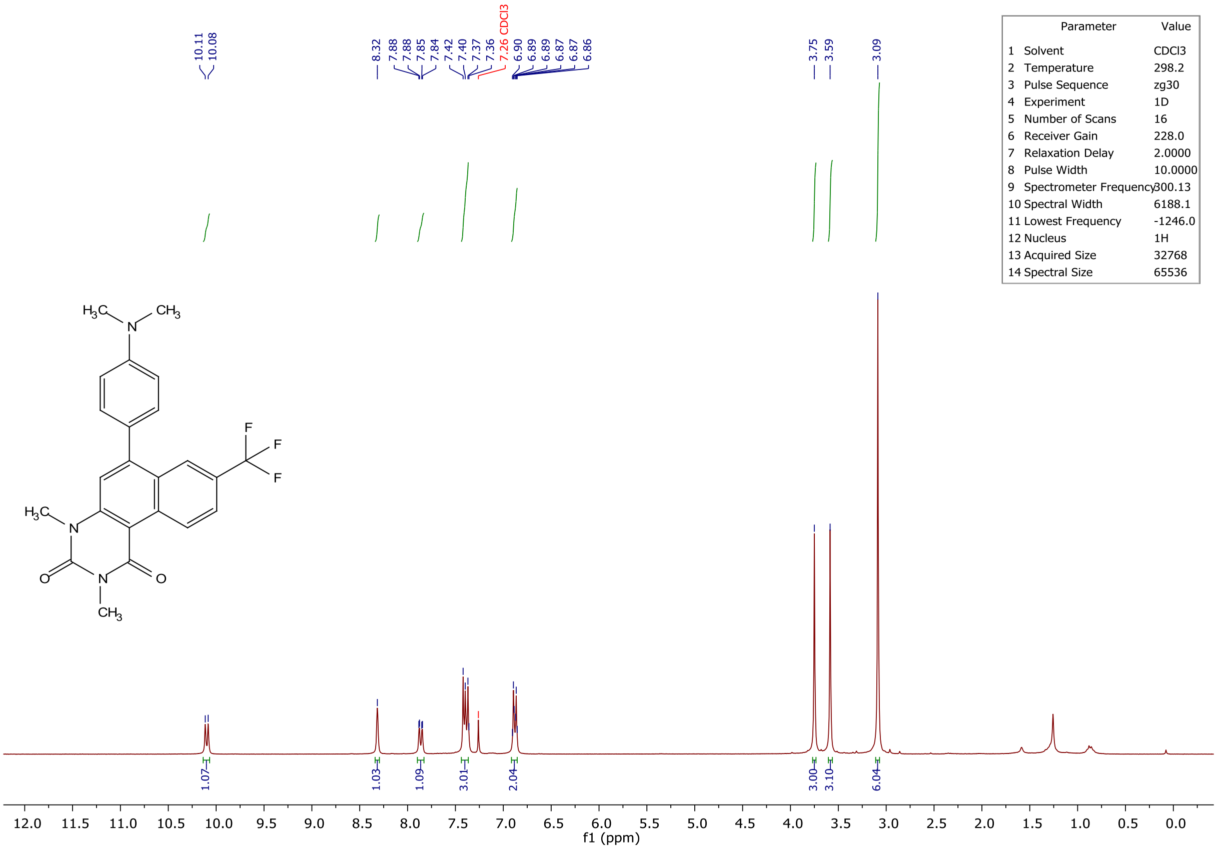


| | Parameter | Value |
|---|---|---|
| 1 | Solvent | CDCl3 |
| 2 | Temperature | 298.2 |
| 3 | Pulse Sequence | zg30 |
| 4 | Experiment | 1D |
| 5 | Number of Scans | 16 |
| 6 | Receiver Gain | 228.0 |
| 7 | Relaxation Delay | 2.0000 |
| 8 | Pulse Width | 10.0000 |
| 9 | Spectrometer Frequency | 300.13 |
| 10 | Spectral Width | 6188.1 |
| 11 | Lowest Frequency | -1246.0 |
| 12 | Nucleus | 1H |
| 13 | Acquired Size | 32768 |
| 14 | Spectral Size | 65536 |

| | Parameter | Value |
|---|---|---|
| 1 | Solvent | CDCl3 |
| 2 | Temperature | 298.2 |
| 3 | Pulse Sequence | zgfhigqn |
| 4 | Experiment | 1D |
| 5 | Number of Scans | 64 |
| 6 | Receiver Gain | 2050.0 |
| 7 | Relaxation Delay | 1.0000 |
| 8 | Pulse Width | 10.0000 |
| 9 | Spectrometer Frequency | 282.38 |
| 10 | Spectral Width | 66964.3 |
| 11 | Lowest Frequency | -61722.4 |
| 12 | Nucleus | 19F |
| 13 | Acquired Size | 65536 |
| 14 | Spectral Size | 131072 |

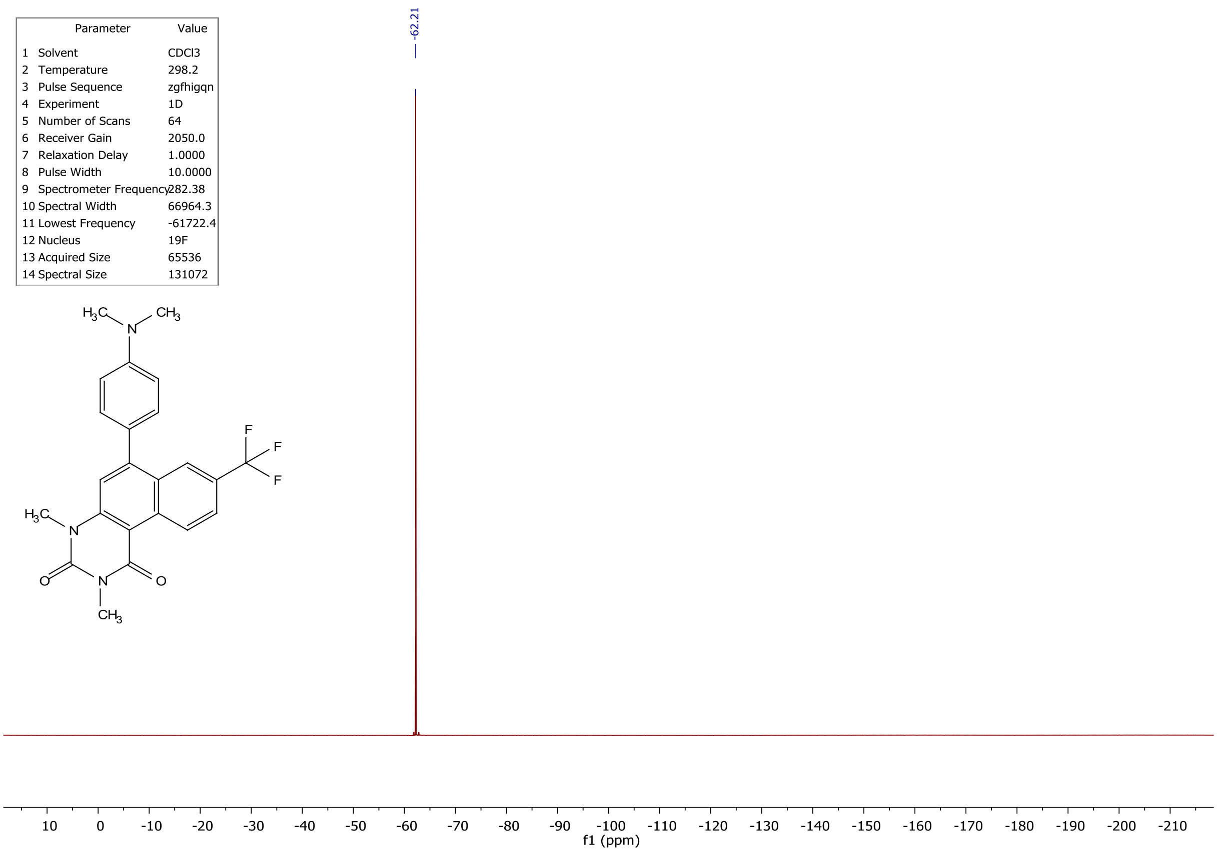

| | Parameter | Value |
|---|---|---|
| 1 | Solvent | CDCl3 |
| 2 | Temperature | 298.2 |
| 3 | Pulse Sequence | zgpg30 |
| 4 | Experiment | 1D |
| 5 | Number of Scans | 1024 |
| 6 | Receiver Gain | 2050.0 |
| 7 | Relaxation Delay | 2.0000 |
| 8 | Pulse Width | 10.0000 |
| 9 | Spectrometer Frequency | 75.48 |
| 10 | Spectral Width | 18028.8 |
| 11 | Lowest Frequency | -1468.1 |
| 12 | Nucleus | 13C |
| 13 | Acquired Size | 32768 |
| 14 | Spectral Size | 65536 |

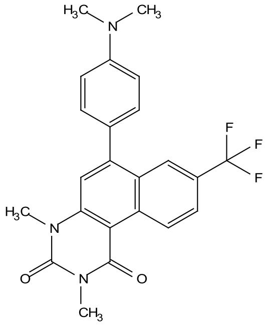


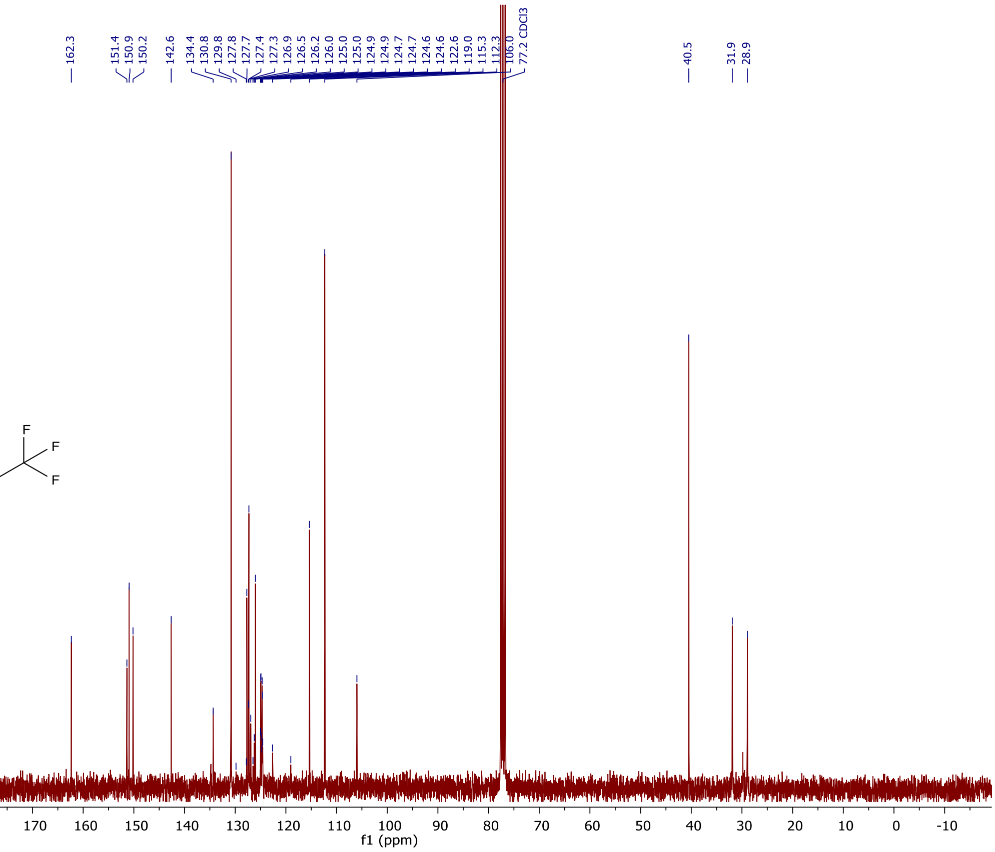

## 2,4-Dimethyl-6-phenylbenzo[*f*]quinazoline-1,3(2*H*,4*H*)-dione (5g)

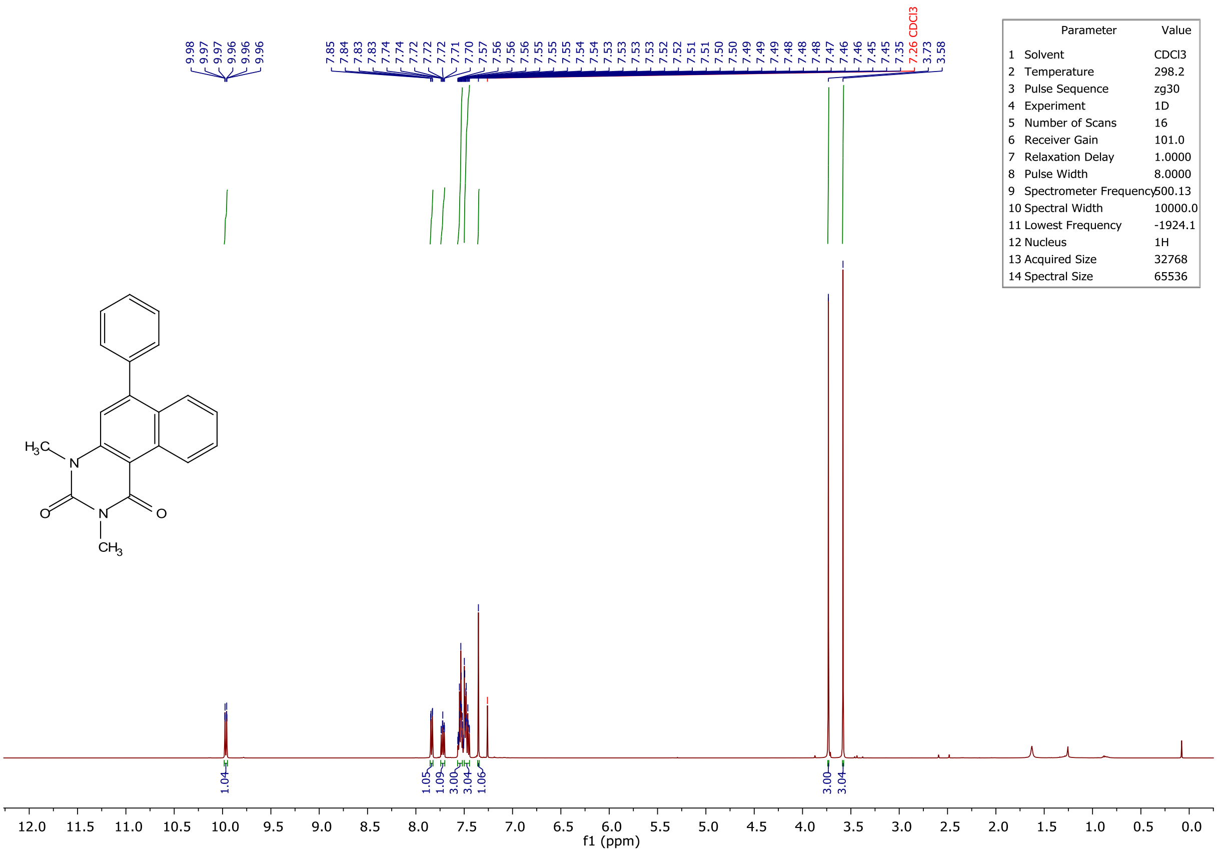


| | Parameter | Value |
|---|---|---|
| 1 | Solvent | CDCl3 |
| 2 | Temperature | 298.2 |
| 3 | Pulse Sequence | zg30 |
| 4 | Experiment | 1D |
| 5 | Number of Scans | 16 |
| 6 | Receiver Gain | 101.0 |
| 7 | Relaxation Delay | 1.0000 |
| 8 | Pulse Width | 8.0000 |
| 9 | Spectrometer Frequency | 500.13 |
| 10 | Spectral Width | 10000.0 |
| 11 | Lowest Frequency | -1924.1 |
| 12 | Nucleus | 1H |
| 13 | Acquired Size | 32768 |
| 14 | Spectral Size | 65536 |

| | Parameter | Value |
|---|---|---|
| 1 | Solvent | CDCl3 |
| 2 | Temperature | 298.2 |
| 3 | Pulse Sequence | zgpg30 |
| 4 | Experiment | 1D |
| 5 | Number of Scans | 1024 |
| 6 | Receiver Gain | 101.0 |
| 7 | Relaxation Delay | 2.0000 |
| 8 | Pulse Width | 10.0000 |
| 9 | Spectrometer Frequency | 125.77 |
| 10 | Spectral Width | 30120.5 |
| 11 | Lowest Frequency | -2470.4 |
| 12 | Nucleus | 13C |
| 13 | Acquired Size | 32768 |
| 14 | Spectral Size | 65536 |

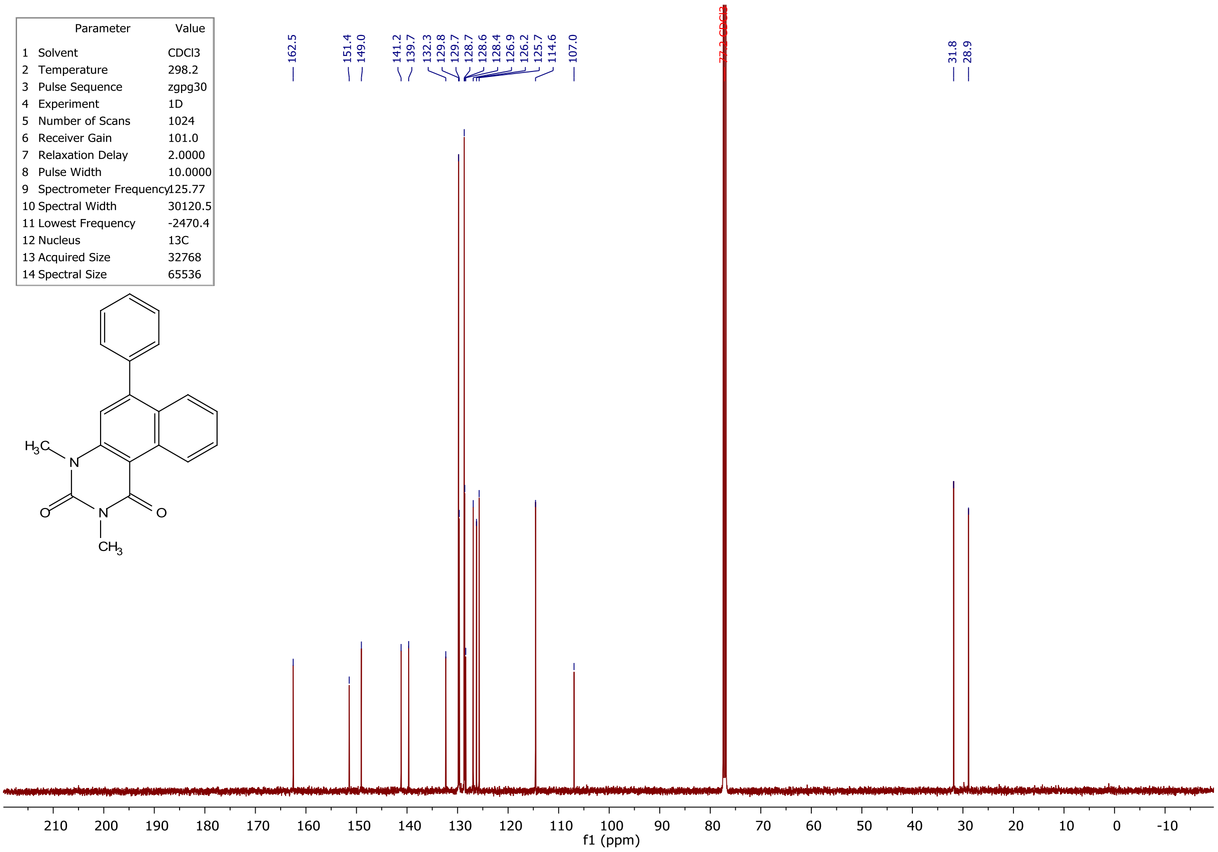

## 6-(4-Fluorophenyl)-8-hydroxy-2,4-dimethylbenzo[*f*]quinazoline-1,3(2*H*,4*H*)-dione (5h)

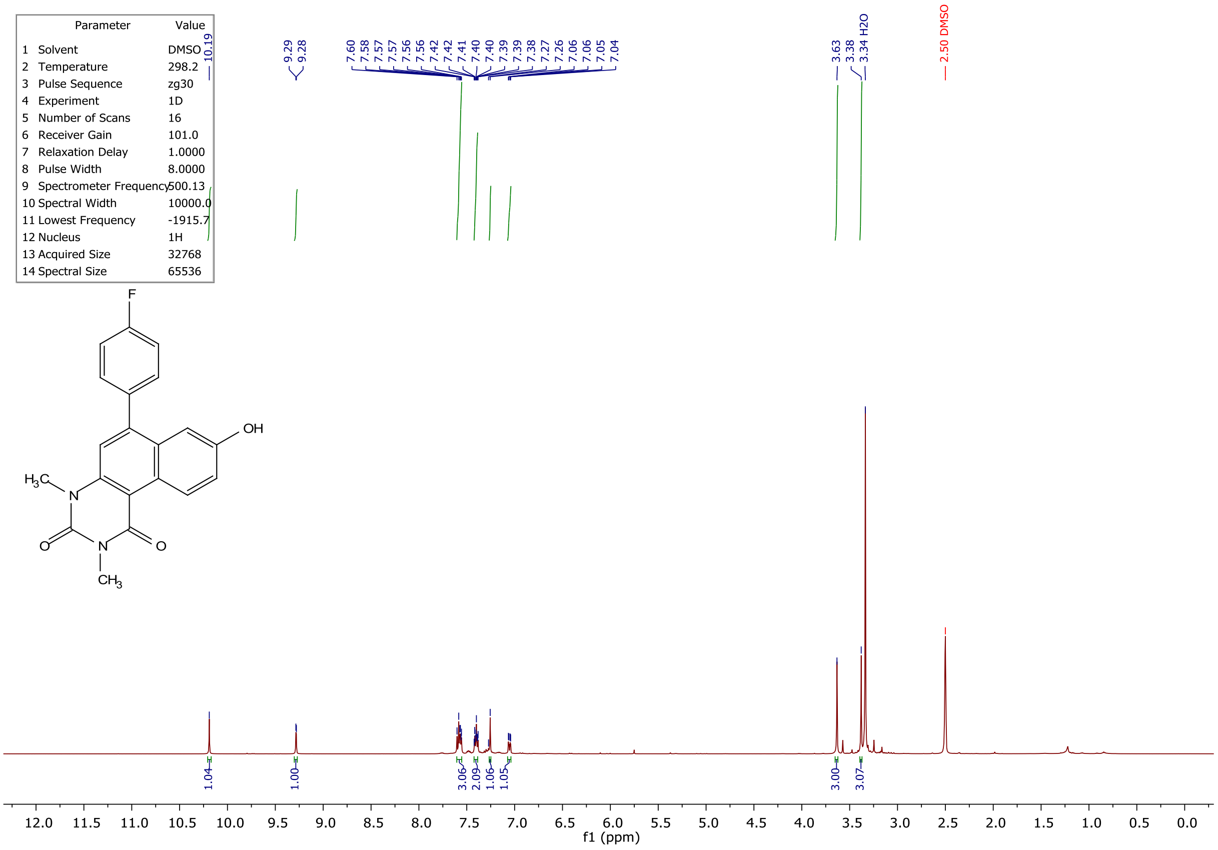


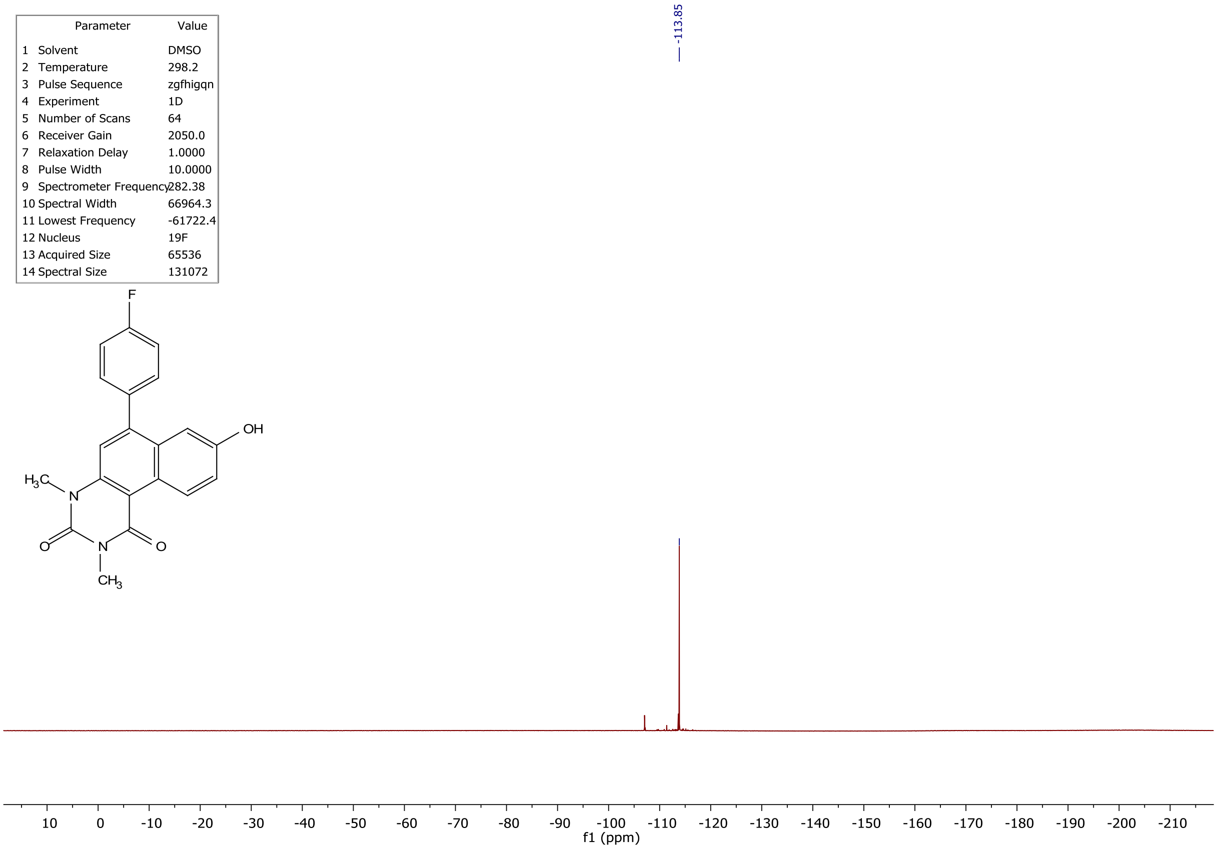

| | Parameter | Value |
|---|---|---|
| 1 | Solvent | DMSO |
| 2 | Temperature | 298.2 |
| 3 | Pulse Sequence | zgpg30 |
| 4 | Experiment | 1D |
| 5 | Number of Scans | 1024 |
| 6 | Receiver Gain | 101.0 |
| 7 | Relaxation Delay | 2.0000 |
| 8 | Pulse Width | 10.0000 |
| 9 | Spectrometer Frequency | 125.77 |
| 10 | Spectral Width | 30120.5 |
| 11 | Lowest Frequency | -2484.5 |
| 12 | Nucleus | 13C |
| 13 | Acquired Size | 32768 |
| 14 | Spectral Size | 65536 |

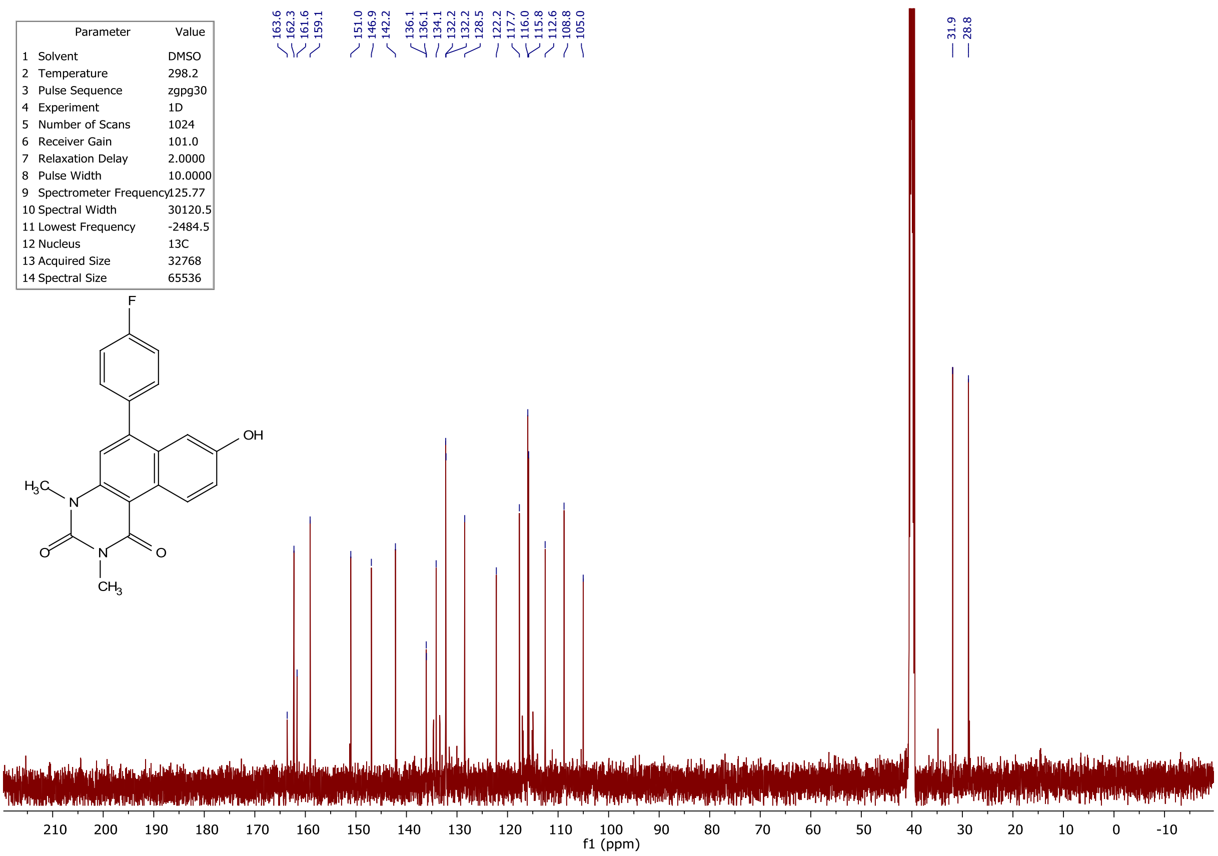

# 2,4-Dimethyl-6-(*p*-tolyl)benzo[4,5]thieno[3,2-*f*]quinazoline-1,3(2*H*,4*H*)-dione (5i)

| | Parameter | Value |
|---|---|---|
| 1 | Solvent | CDCl3 |
| 2 | Temperature | 298.2 |
| 3 | Pulse Sequence | zg30 |
| 4 | Experiment | 1D |
| 5 | Number of Scans | 16 |
| 6 | Receiver Gain | 101.0 |
| 7 | Relaxation Delay | 1.0000 |
| 8 | Pulse Width | 8.0000 |
| 9 | Spectrometer Frequency | 500.13 |
| 10 | Spectral Width | 10000.0 |
| 11 | Lowest Frequency | -1924.1 |
| 12 | Nucleus | 1H |
| 13 | Acquired Size | 32768 |
| 14 | Spectral Size | 65536 |

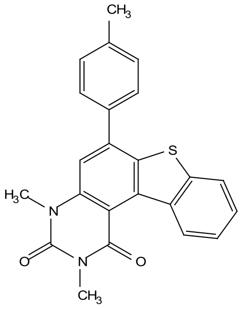


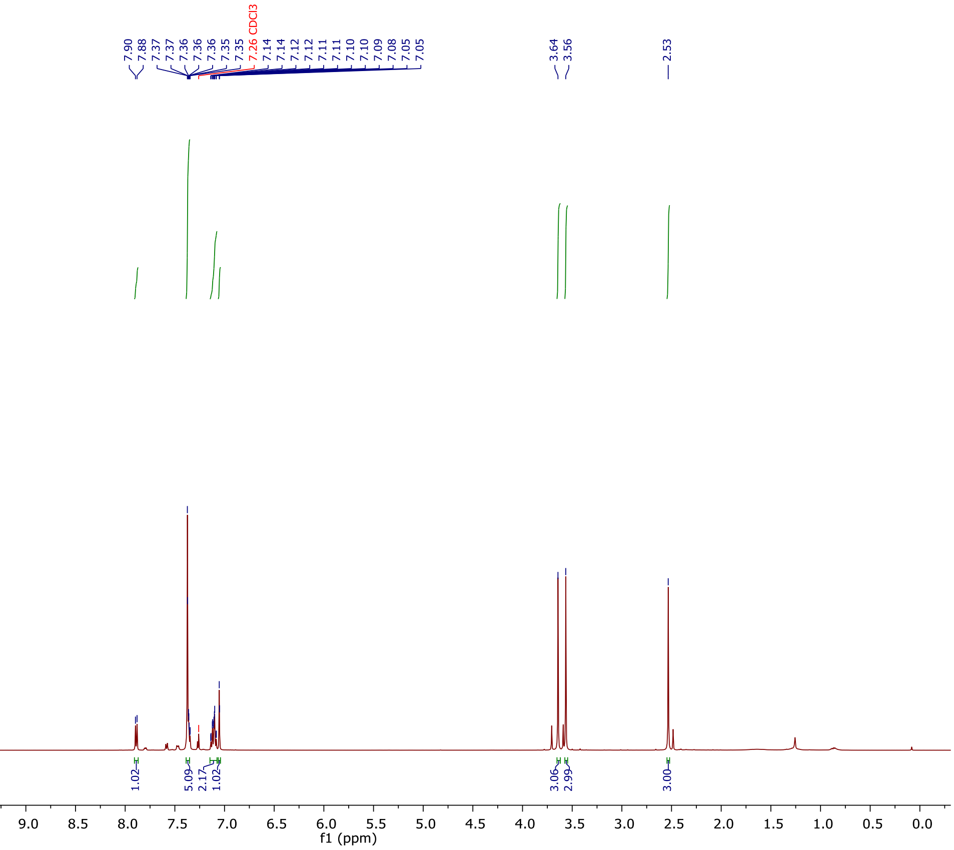


| | Parameter | Value |
|---|---|---|
| 1 | Solvent | CDCl3 |
| 2 | Temperature | 298.1 |
| 3 | Pulse Sequence | zgpg30 |
| 4 | Experiment | 1D |
| 5 | Number of Scans | 4096 |
| 6 | Receiver Gain | 101.0 |
| 7 | Relaxation Delay | 2.0000 |
| 8 | Pulse Width | 10.0000 |
| 9 | Spectrometer Frequency | 125.77 |
| 10 | Spectral Width | 30120.5 |
| 11 | Lowest Frequency | -2471.4 |
| 12 | Nucleus | 13C |
| 13 | Acquired Size | 32768 |
| 14 | Spectral Size | 65536 |

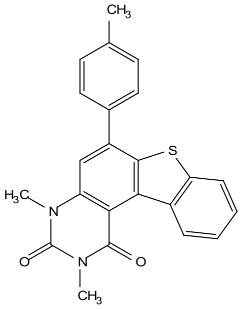


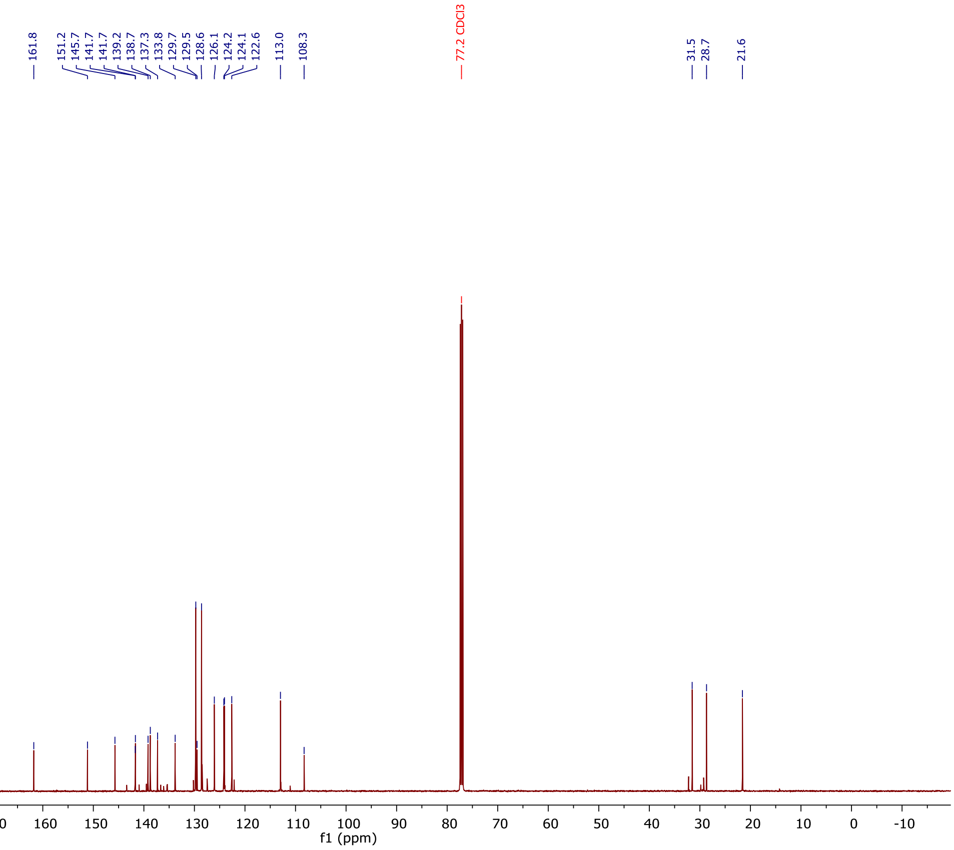

Supplement: File 1 — Copies of NMR spectra. [file Beilstein_J_Org_Chem-20-2708-s001.pdf]
